# Machine Learning Potential for Calcium Silicate Hydrates With Broad Compositional and Structural Diversity

**DOI:** 10.1002/advs.77942

**Published:** 2026-09-28

**Authors:** Yunjian Li, Cheng Chen, Roland J.‐M. Pellenq, Zongjin Li, Jiaping Liu

**Affiliations:** ^1^ State Key Laboratory of Climate Resilience for Coastal Cities, Department of Civil and Environmental Engineering The Hong Kong University of Science and Technology Hong Kong People's Republic of China; ^2^ Institut Européen des Membranes CNRS Université De Montpellier Montpellier France; ^3^ Faculty of Innovation Engineering Macau University of Science and Technology Taipa Macao People's Republic of China; ^4^ School of Materials Science and Engineering Southeast University Nanjing People's Republic of China

**Keywords:** atomistic modeling, calcium silicate hydrate, cement, machine learning potential, mechanical property, nanostructure

## Abstract

Calcium silicate hydrate (C–S–H), the primary hydration product of cement, governs the strength and durability of concrete but remains difficult to model due to its chemical heterogeneity and disordered nanostructure. Machine learning potentials (MLPs) offer a route to extend quantum accuracy to large‐scale simulations, yet progress has been limited by the absence of representative datasets. Here, we present a stoichiometry‐generalizable MLP, CSH‐MLP, trained on a broad and representative dataset containing over 50 000 DFT‐labeled configurations, which is derived from nearly 700 distinct C–S–H compositions spanning diverse compositions, defects, and hydration states. CSH‐MLP reproduces structural, dynamic, and mechanical properties with near‐DFT accuracy, resolves long‐standing discrepancies with high‐pressure X‐ray diffraction, and transfers reliably to large, disordered, and nanoporous models beyond its training set. Achieving substantially higher computational efficiency than ReaxFF, CSH‐MLP enables simulations at scales and timescales previously inaccessible to quantum‐mechanical methods. This work establishes a foundation for multiscale cement modeling, data‐driven “cement genome” construction, and design of next‐generation sustainable construction materials.

## Graphical abstract

Existing machine learning potentials remain limited in their ability to generalize across the broad chemical and structural variability of C–S–H. Here, a representative DFT‐labeled dataset is constructed through large‐scale model generation, redundancy‐controlled screening, first‐principles optimization, and targeted sampling of non‐equilibrium configurations, providing a robust foundation for stoichiometry‐generalizable, quantum‐accurate simulations of cementitious materials.

## 1 Introduction

Cement, a material with origins dating back over 2000 years and industrially developed more than 200 years ago [1], is the most widely produced material by humankind. However, it is also a major contributor to global carbon emissions, accounting for nearly 8% of the total [2, 3]. Reducing the environmental footprint of cement while meeting the rising demand for infrastructure requires a deep, predictive understanding of its performance across length scales [4, 5]. The central binding phase in hydrated cement paste is calcium silicate hydrate (C–S–H), a nanocrystalline and X‐ray amorphous gel with variable composition and local disorder, which is primarily responsible for the cohesion, strength, and durability of cementitious materials [6]. Despite its critical role, the structure and mechanical behavior of C–S–H remains elusive at the atomic scale, largely due to its heterogeneous chemistry and poorly defined crystallinity. This complexity has long frustrated efforts to experimentally resolve its local structure and predict its properties.

Atomistic simulations, particularly molecular dynamics (MD) and ab initio calculations, have proven invaluable in elucidating the structural, mechanical, and thermodynamic properties of cement, as well as providing critical insights into atomic‐scale arrangements, defect structures, hydration dynamics, and interfacial interactions, which complement experimental observations [7, 8, 9, 10, 11]. Ab initio methods, such as density functional theory (DFT), offer superior accuracy and are widely regarded as the benchmark for property prediction. However, their computational cost limits their applicability to small systems and short timescales, impeding comprehensive investigations of complex, disordered materials [12, 13]. In contrast, classical MD simulations allow for larger spatial and temporal scales, but their reliability depends heavily on the quality of force fields.

Several force fields have been developed for cement modeling, each with inherent trade‐offs. ClayFF is widely used due to its broad elemental coverage and low computational cost, but it was originally parameterized for clay minerals and struggles to reproduce the mechanical properties of C–S–H [14]. The CSH‐FF, specifically tailored for C–S–H, improves upon this by fitting to ab initio data for crystalline tobermorite but remains an empirical model incapable of capturing reactive chemistry [15]. ReaxFF introduces bond‐breaking and dynamic reactivity, making it suitable for modeling hydration and hydroxylation processes during the initial cement hydration [16]. Nevertheless, it is parameter‐dependent for certain kinds of chemical reactions and suffers from poor mechanical accuracy and high computational demand [17, 18]. ERICA FF, based on the Cement FF framework [19], supports a broader range of cement‐relevant elements and has shown improved agreement with DFT predictions in bulk modulus and structural predictions [20]. But it remains limited by its Lennard‐Jones and Buckingham potential mix.

To address the spatial and temporal limitations of quantum mechanical simulations while retaining their accuracy, machine learning potentials (MLPs) or machine learning interatomic potentials (MLIPs), have emerged as a powerful alternative. MLPs enable near‐DFT accuracy at dramatically reduced computational cost, allowing simulations of large and disordered systems over nanosecond timescales at a fraction of the cost of conventional ab initio methods [18, 21, 22, 23, 24, 25, 26, 27, 28]. Recent studies have begun to explore the potential of MLPs in cementitious systems. For example, Zhou et al. trained an MLP specifically for tobermorite structures and validated its accuracy against ab initio molecular dynamics (AIMD) simulations [26]. Building on this, Li et al. later incorporated C–S–H structures with varying calcium‐to‐silicon (Ca/Si) ratios into the training data, enabling the exploration of mechanical properties, but still within a constrained structural regime [18, 27]. Li et al. developed an MLP to investigate the thermodynamics and kinetics of dicalcium silicate dissolution across different temperatures by iteratively constructing the training dataset to achieve robust performance [28]. Jiang et al. fine‐tuned the DPA‐2 model [29] to build an MLP for exploring hydrogel‐Ca reaction pathways [30]. Beyond cementitious systems, recent chemistry‐specific MLPs have demonstrated DFT‐level accuracy in predicting multiple properties and enabled large‐scale molecular dynamics [31, 32, 33]. For C–S–H, however, existing MLPs remain constrained by relatively narrow compositional and configurational spaces, which limits their applicability to its highly heterogeneous structures. The key challenge, therefore, lies in constructing a representative dataset that spans the full chemical and structural diversity of C–S–H, including variations in composition, defect structures, and thermodynamic states, and in developing an MLP capable of generalizing across this broad configurational landscape.

In this study, we introduce a data‐generation strategy to construct a C–S–H atomic‐structure dataset, which spans a broad range of Ca/Si ratios, defect topologies, and hydration states. Based on the C–S–H dataset, we train an accurate and generalizable MLP for C–S–H, CSH‐MLP, and validate its accuracy by benchmarking structural and mechanical predictions against ab initio simulations and comparison between previously developed force fields. The principal advance of this work lies in the diversity of the C–S–H dataset and the resulting compositional and structural generalizability of CSH‐MLP. The proposed CSH‐MLP not only shows high fidelity in predicting the mechanical properties and structural features of C–S–H, but also achieves stoichiometry‐generalizability across nearly 700 distinct compositions, scalability to large‐scale systems, and remarkable extrapolative capability to unseen structures. Notably, CSH‐MLP reconciles long‐standing discrepancies between high‐pressure X‐ray diffraction measurements and defect‐based C–S–H models. We further show that CSH‐MLP achieves up to approximately 30‐fold computational speedup over ReaxFF, enabling large‐scale simulations of cementitious materials with near‐DFT accuracy. These contributions address a critical gap in the atomic modeling of cementitious materials and will enable accurate, scalable simulations of C–S–H and related systems at unprecedented levels of detail.

## 2 Results

### 2.1 Dataset Construction

An accurate and generalizable machine learning potential (MLP) is heavily dependent on the quality and diversity of the training dataset. However, to date, no publicly available database contains experimentally derived atomic structures of cement due to its nanocrystalline nature, ​heterogeneous compositions, and dynamic chemical environments. These challenges make cement a particularly difficult material to model comprehensively using computational methods. To fill this gap, we developed a data‐generation strategy for constructing a DFT‐labeled dataset that captures the unique characteristics of C–S–H structures. The data‐generation strategy proposed in this work includes four main steps: (i) model construction and redundancy control, (ii) structure optimization, (iii) non‐equilibrium data augmentation, and (iv) DFT single‐point calculations (Figure 1).

**FIGURE 1:**
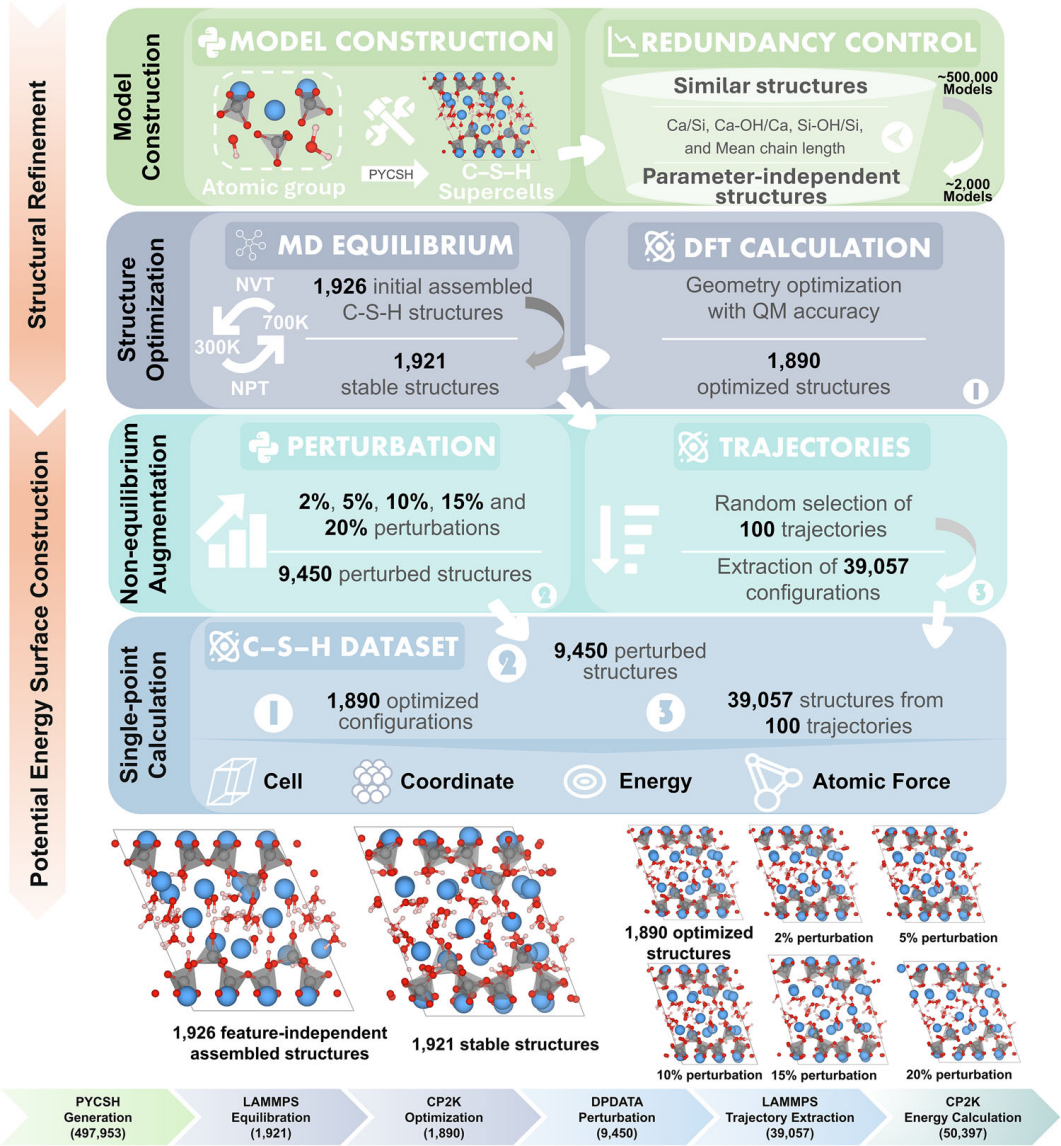
Schematic diagram of data‐generation strategy for C–S–H dataset. The flowchart includes four main steps: (i) model construction and redundancy control, (ii) structure optimization, (iii) non‐equilibrium data augmentation, and (iv) DFT single‐point calculations. In the first stage, ∼500 000 C–S–H supercells were generated by the pyCSH program, and a feature‐independent screening process reduced these to 1926 parameter‐independent structures spanning 688 compositions. In the second stage, the selected models were first equilibrated using quenching molecular dynamics (MD) simulations under NVT (canonical ensemble) and NPT (isothermal‐isobaric ensemble) ensembles, and subsequently optimized at the density functional theory (DFT) level. This results in a total of 1890 configurations. In the third stage, a two‐step augmentation strategy that includes structural perturbations (9450 perturbed variants) and structure harvesting from 100 MD equilibration trajectories (39 057 configurations) was adopted to sample non‐equilibrium and metastable configurations. All configurations were labeled with high‐accuracy DFT single‐point calculations, constituting the final C–S–H dataset.

In the first stage, to construct a comprehensive configurational space that captures the atomic and structural diversity of C–S–H, we initially generated approximately 500 000 independent and unique 2 × 2 × 1 C–S–H supercells with calcium‐to‐silicon (Ca/Si) ratios ranging from 0.83 to 2.5 using the pyCSH program. Among these, 2047 supercells were identified as duplicates, indicating that the configurational space defined by the pyCSH construction rules had been sampled in a sufficiently exhaustive manner. These models were generated using an algorithmic approach to incorporate key structural and compositional features, including the Ca/Si ratio, silanol to silicon (Si─OH/Si) ratio, calcium‐hydroxyl to calcium (Ca─OH/Ca) ratio, and mean chain length (MCL). To minimize dataset redundancy while preserving a well representation of the configurational space that retains the full structural and compositional diversity of C–S–H systems, we screened each C–S–H supercell based on four key parameters (Ca/Si, Si─OH/Si, Ca─OH/Ca, and MCL) and randomly selected up to three configurations per parameter set. Figure S10 illustrates the interrelationships among these parameters, demonstrating that the bulk C–S–H models in our dataset not only adhere to experimentally observed statistical trends but also span a wide parameter space. This extensive coverage ensures a comprehensive representation of the configurational space. In addition, we imposed an additional constraint to preserve the linear correlation between Ca/Si and (H_2_O + 0.5OH^−^)/Si (2H/Si) ratios, thereby ensuring consistency with experimental observations [6]. As a result, 1926 C–S–H models covering 688 compositions with distinct compositional and structural variations were obtained.

Subsequently, the selected C–S–H models were equilibrated through molecular dynamics (MD) simulations using the ERICA FF, resulting in 1921 MD‐stable configurations along with their corresponding equilibration trajectories. We should note that five unphysical structures collapsed during the equilibration process, which is well below the 5% collapse rate reported by the pyCSH developers [34]. Then, these MD‐stable models were further optimized by density functional theory (DFT) calculations, which enables accurate sampling of different local minima on the potential energy surface (PES). After excluding configurations that failed to converge, a total of 1890 DFT‐optimized configurations were obtained, which constitute a core part of the final dataset.

Developing a robust MLP for cementitious materials requires training data that includes not only energy minima, but also transition and metastable states on the PES. To meet this requirement, we adopted a two‐stage dataset augmentation strategy. First, structural perturbations with displacement magnitudes of 2%, 5%, 10%, 15%, and 20% were applied to all atoms of the DFT‐optimized models by deforming the entire simulation cell while keeping fractional atomic coordinates fixed. This process generates 9450 perturbed configurations, which will improve the robustness of the MLP when the system is at high‐energy, non‐equilibrium states.

Second, additional atomic configurations were extracted from multiple MD‐equilibrated trajectories. Typically, configurations derived from MD trajectories for MLP development are obtained from a limited set of models, which constrains the generalizability of the MLP. To overcome this limitation, we expanded the training dataset by randomly selecting 100 MD trajectories from 1921 MD‐equilibration trajectories with an insurance of a uniform distribution of Ca/Si ratios ranging from 0.88 to 2.44. Following the strategy described in Section S2.1, a total of 39 057 configurations were added to the dataset. As a result, more than 50 000 configurations were collected from the process of DFT‐optimization, perturbation, as well as MD‐equilibration. These configurations were then labeled by DFT single‐point calculations with higher precision and serve as the training and validation dataset for the MLP of C–S–H in this work, hereafter referred to as CSH‐MLP.

### 2.2 Structural Analysis of C–S–H Dataset

The structural diversity within the C–S–H dataset was identified by the smooth overlap of atomic positions (SOAP) fingerprinting method [35], and the structural features of the SOAP descriptor were projected onto a two‐dimensional plane by the principal component analysis (PCA) dimensionality reduction algorithm. Figure 2 captures the inherent structural information across different sub‐datasets, including C–S–H structures after DFT optimization, perturbation, and those extracted from the MD trajectories. The results exhibit a high degree of dispersion, confirming the effectiveness of the proposed screening method in capturing a broad spectrum of configurational space. Moreover, the Ca/Si and 2H/Si ratios have a significant impact on the structural parameters of the dataset. In the optimization sub‐dataset, the data are positively distributed along the PCA‐1 axis as both the Ca/Si and 2H/Si ratios increase (Figure 2a,b). By contrast, the perturbation and trajectory sub‐datasets show a positive shift along PCA‐2 as the Ca/Si and 2H/Si ratios increase (Figure 2d,e,g,h). The synchronous upward trend of the Ca/Si and the 2H/Si is in good agreement with the experimental results [6]. In Figure 2a,b, the colors distinctly differentiate various features. Meanwhile, the changes in the ratios are continuous. However, compared with the clear changes in the 2H/Si ratio, although the changes in the Ca/Si ratio are mainly continuous, there are some interlacing situations. This indicates that among the structures with the same Ca/Si ratio, there are cases of different water contents in our dataset. While maintaining the relationships of objective physical laws, it adds more reasonable possibilities.

**FIGURE 2:**
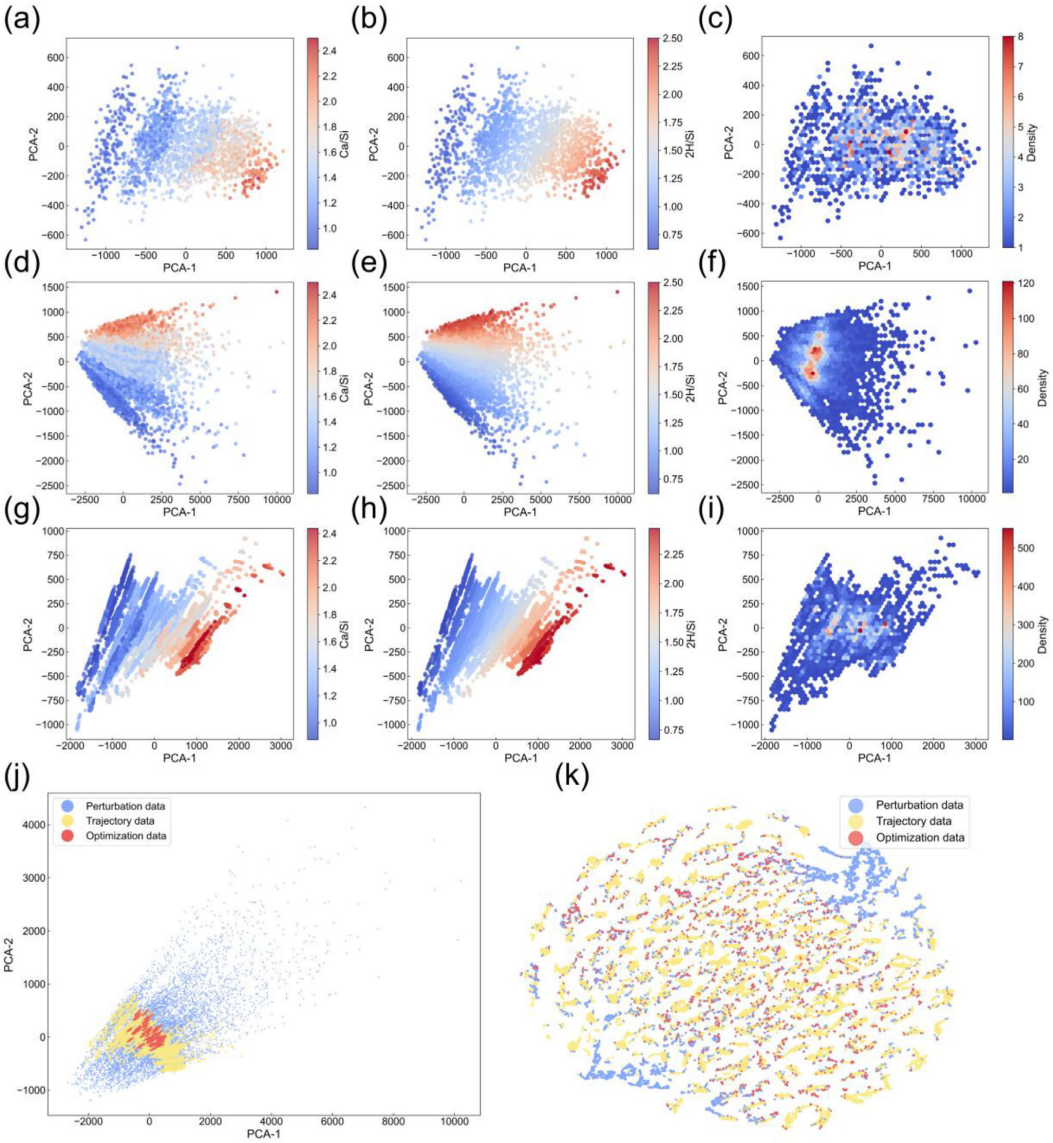
Distributions of training data in reduced‐dimensional space. (a–c) Optimization data distributions after dimensionality reduction by principal component analysis (PCA), with color mappings representing Ca/Si, 2H/Si, and data density, respectively. (d–f) Perturbation data distributions after dimensionality reduction by PCA, with color mappings representing Ca/Si, 2H/Si, and data density, respectively. (g–i) Trajectory data distributions after dimensionality reduction by PCA, with color mappings representing Ca/Si, 2H/Si, and data density, respectively. (j) Combined PCA distributions of all datasets. ‘Optimization data’ refers to configurations after DFT optimization, ‘Perturbation data’ refers to configurations after structural perturbation. ‘Trajectory data’ correspond to configurations randomly selected from MD trajectories. (k) Combined distributions of all datasets after t‐distributed stochastic neighbor embedding (t‐SNE) reduction, showing the overlap and complementarity of the three data types.

The density maps of all sub‐datasets exhibit a clear outward spread from the origin (Figure 2c,f,i), indicating a decreasing similarity in configurations as the distance from the origin increases. Figure 2j shows the distribution of the data after merging the three sub‐datasets. The optimization sub‐dataset is distinctly centered at the origin, suggesting that the optimized C–S–H structures have high structural similarity, which provides strong support for the MLP to optimize the system to a stable state. The trajectory sub‐dataset, on the other hand, covers a slightly broader configurational space, reflecting the involvement of more complex and variable configurations during the optimization process. The perturbation sub‐dataset shows an extremely decentralized and broad distribution of configurations, which enhances the robustness of the MLP when the system encounters extreme configurations during MD simulations. Further analysis using t‐distributed stochastic neighbor embedding (t‐SNE) dimensionality reduction confirms that the dataset preparation in this study effectively captures the full range of possible configurations within the C–S–H system (Figure 2k).

### 2.3 Validation of the C–S–H Machine Learning Potential

The accuracy of the CSH‐MLP was validated by comparing the atomic energy and forces predicted by the MLP with those calculated using density functional theory (DFT). Figure 3 presents a strong linear correlation between the MLP‐predicted and DFT‐calculated atomic energies and forces for both training and test datasets. Given that our dataset comprises 688 compositions, it exceeds the scale of many previously used datasets, thereby enhancing the broader applicability of the CSH‐MLP. The root mean squared errors (RMSE) for atomic energies are 1.18 and 1.50 meV/atom for the training and test datasets, respectively. For atomic forces, the RMSE values for the training and test datasets are 0.092 and 0.102 eV/Å, respectively. According to the previous developed MLPs [18, 26, 28, 36, 37, 38, 39, 40], these values fall within the reasonable ranges of 0.5–6.5 meV/atom for energy errors, and 60—200 meV/Å for force errors, placing CSH‐MLP toward the lower end of both. This confirms that CSH‐MLP attains a high level of accuracy and robustness for C–S–H simulations. Distributions of the absolute errors were provided in Figure S3.

**FIGURE 3:**
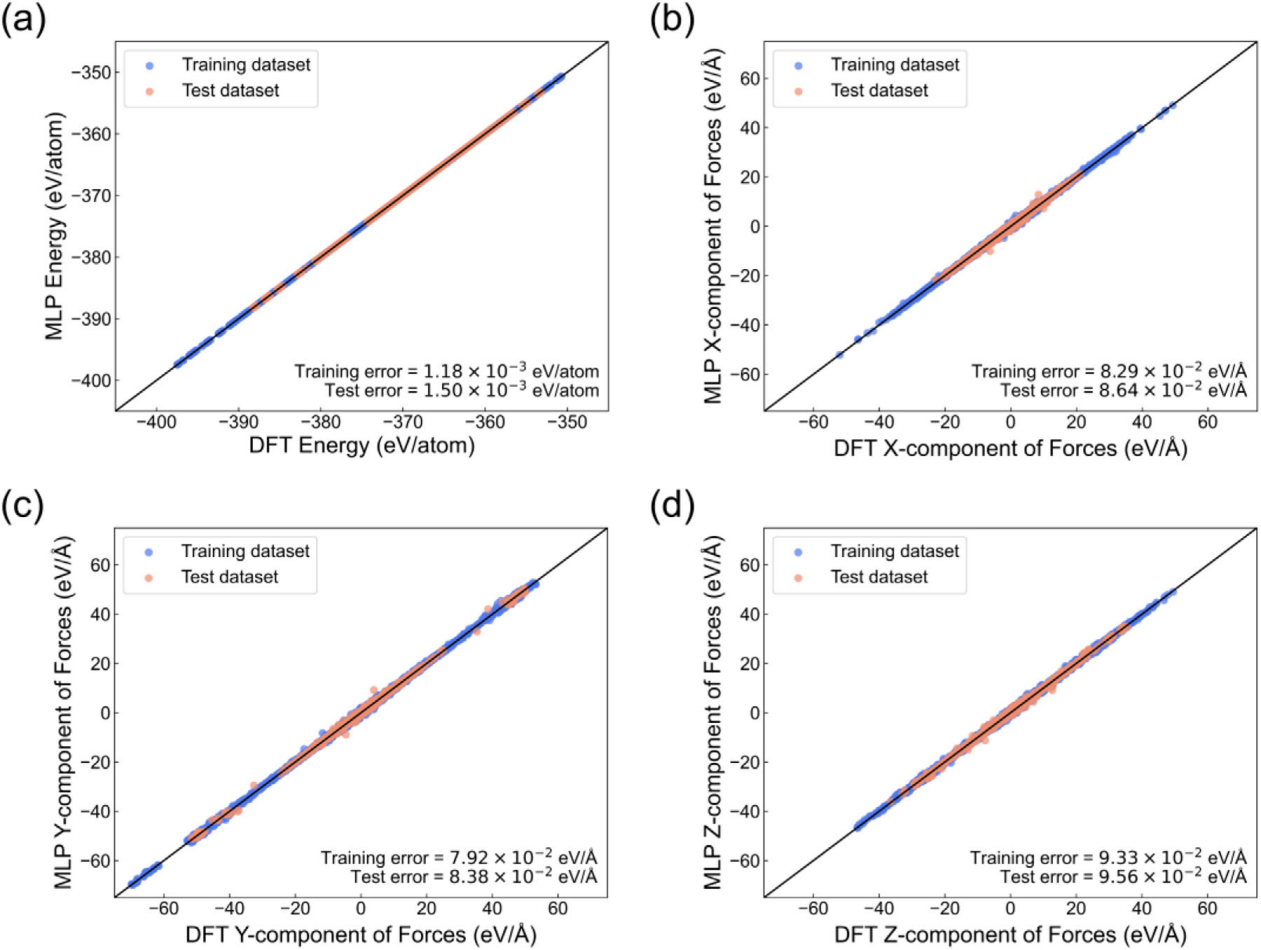
Comparison of the atomic energies and forces calculated by the MLP and DFT for configurations in the final training dataset. (a–d) MLP *vs* DFT results for energies, x‐component of forces, y‐component of forces, and z‐component of forces, respectively. The training dataset comprises 47 867 configurations, while the test dataset contains 2530 configurations. The reported “error” refers to the root mean square error (RMSE).

To further evaluate whether this accuracy is retained for configurations sampled during machine‐learning molecular dynamics (MLMD) simulations, we further benchmarked the Ca/Si = 1.47 model in Figure 4a against DFT. As shown in Figure S6, CSH‐MLP reproduces the reference atomic forces with an RMSE of 1.07 × 10^−1^ eV/Å. These results demonstrate that CSH‐MLP maintains consistent accuracy in the production simulations.

**FIGURE 4:**
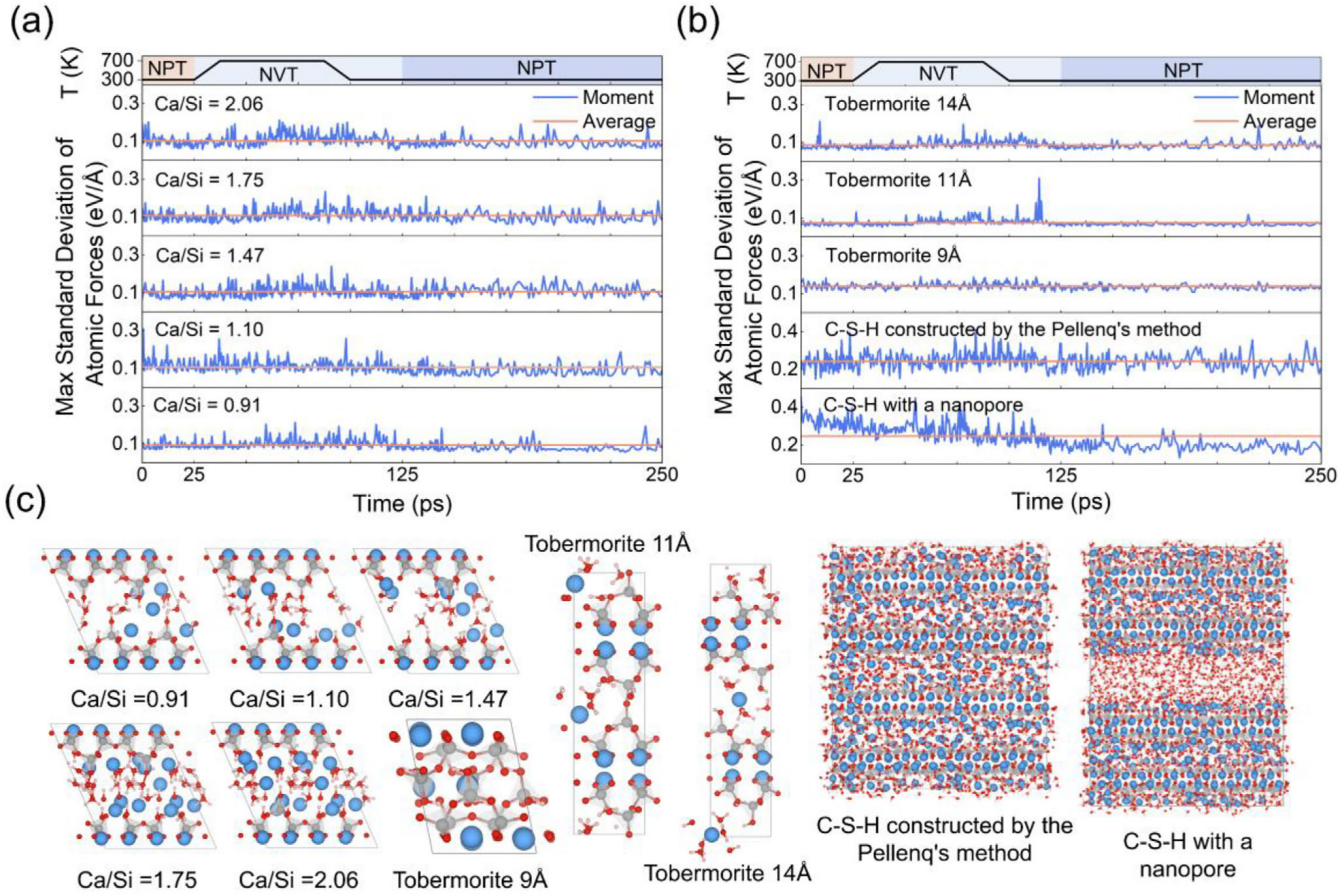
Assessment of reliability of CSH‐MLP during machine‐learning molecular dynamics simulations. (a) Maximal standard deviation of atomic forces for five C–S–H models spanning different Ca/Si ratios, evaluated under combined NVT and NPT ensembles. (b) Maximal standard deviation of atomic forces for two C–S–H models (large‐scale bulk C–S–H model containing 8456 atoms and C–S–H gel model with 10 109 atoms) constructed using the Pellenq's method [8], and three tobermorite models of different basal spacings [41, 42, 43], evaluated under combined NVT and NPT ensembles. (c) Schematic of the atomic models used in the simulations. Blue sphere—Ca; grey sphere—Si; red sphere—O and white sphere—H.

To assess the reliability of CSH‐MLP during machine‐learning molecular dynamics (MLMD) simulations, we monitored the maximum standard deviation of the predicted atomic forces (ε_*F*_) for five distinct C–S–H models with Ca/Si ratios ranging from 0.91 to 2.06. As shown in Figure 4a, the values of ε_*F*_ across different C–S–H compositions are consistently low during both NPT equilibration and the subsequent NVT quenching process, with an average value of approximately 0.1 eV/Å and frequency distribution centered below 0.1 eV/Å (Figure S1).

Further verification of the robustness of the dataset was conducted using C–S–H structures outside the training dataset (Figure 4b, Figure S2). We chose two large‐scale C–S–H models with a Ca/Si ratio of 1.7 reported by Duque‐Redondo et al. [44], which were originally constructed using the method of Pellenq et al. [8]. One model corresponds to a bulk C–S–H structure containing 8456 atoms, while the other represents a C–S–H gel model with 10 109 atoms, including a ∼1 nm nanopore filled with water. CSH‐MLP successfully and stably equilibrates both C–S–H models during MLMD simulations, demonstrating the strong generalizability of the developed CSH‐MLP to the C–S–H model.

Because mechanical testing is critical for validating the practical applicability of CSH‐MLP, ε_*F*_ was monitored during MLMD simulations of uniaxial tensile deformation along the x, y, and z directions. As shown in Figure S5, ε_*F*_ remains consistently low and stable throughout the simulations, even under strains reaching up to 100%.

### 2.4 Benchmarking of C–S–H Structural Features

The lattice constants of tobermorite structures with varying interlayer spacings and the C–S–H model were optimized using both MLMD and DFT simulations, and the results were compared with those obtained from previously developed force fields, as summarized in Table 1. For the C–S–H benchmark model, CSH‐MLP demonstrates superior performance, with a maximum deviation of only 0.23% and an average discrepancy of merely 0.14%, significantly outperforming the other force fields. Notably, tobermorite configurations were not included in the training dataset. The largest deviation between CSH‐MLP and DFT predictions occurs for 14 Å tobermorite, amounting to only 1.72%. For other tobermorite structures, the errors remain below 1%, demonstrating the excellent applicability of CSH‐MLP across the tobermorite family. These results underscore the outstanding capability of CSH‐MLP in accurately reproducing the structural characteristics of nanocrystalline C–S–H‐like phases. Although csh_cp achieves slightly better accuracy than CSH‐MLP in predicting the 14 Å tobermorite structure, this result is reasonable and acceptable because csh_cp was primarily developed based on tobermorite equilibrium trajectories, whereas CSH‐MLP has never been exposed to such configurations during training.

**TABLE 1:** Lattice constants of the C–S–H benchmark model and tobermorite phases (9, 11, and 14 Å) as obtained by DFT, CSH‐MLP, csh_cp, ReaxFF, ClayFF, and MACE‐MP‐0. Tobermorite data, except for CSH‐MLP, were taken from previous work [45]. Mean discrepancy denotes the deviation of each force field prediction from the corresponding DFT reference values.

		a(Å)	b(Å)	c(Å)	α(°)	β(°)	γ(°)	Mean Discrepancy (%)
C–S–H benchmark model	DFT	13.19	14.71	13.85	107.95	94.85	123.74	
	CSH‐MLP	13.17	14.67	13.83	107.97	95.00	123.54	**0.14**
	csh_cp	13.24	14.71	14.27	106.80	98.48	123.14	1.47
	ReaxFF	13.07	14.81	13.84	106.84	95.82	124.18	0.68
	ClayFF	13.50	14.74	13.75	109.66	88.26	124.40	2.06
	MACE‐MP‐0	13.28	14.84	14.32	107.61	96.92	123.13	1.32
Tobermorite 9Å	DFT	11.18	7.33	9.48	99.04	92.08	90.68	
	CSH‐MLP	11.24	7.31	9.47	99.02	91.99	90.77	**0.16**
	csh_cp	11.37	7.34	9.56	99.29	89.56	90.37	1.00
	ReaxFF	10.88	7.33	9.78	101.12	93.55	90.52	1.63
	ClayFF	11.29	7.29	9.70	98.04	88.61	89.40	1.66
	MACE‐MP‐0	11.36	7.39	9.70	99.43	90.87	90.23	1.15
Tobermorite 11Å	DFT	6.79	7.43	22.52	90.07	89.70	123.23	
	CSH‐MLP	6.77	7.38	22.56	90.35	89.43	122.99	**0.30**
	csh_cp	6.84	7.44	22.82	90.08	88.57	122.87	0.64
	ReaxFF	6.59	7.35	23.57	90.75	89.46	123.30	1.61
	ClayFF	6.75	7.33	22.52	90.01	89.79	122.70	0.41
	MACE‐MP‐0	6.85	7.47	22.88	90.02	89.91	122.96	0.59
Tobermorite 14Å	DFT	6.74	7.47	27.13	89.76	90.17	122.84	
	CSH‐MLP	6.69	7.38	26.86	89.13	91.72	121.79	**1.03**
	csh_cp	6.80	7.42	26.94	88.26	91.15	122.29	0.92
	ReaxFF	6.55	7.45	28.34	90.04	89.91	123.42	1.43
	ClayFF	6.77	6.76	27.46	90.21	89.06	114.27	3.31
	MACE‐MP‐0	6.81	7.46	28.31	90.00	86.49	123.10	1.69

General‐purpose machine learning potentials (MLPs) are typically trained on extensive datasets of inorganic/organic materials covering more than 80 elements, making them broadly accessible and seemingly convenient to apply across diverse systems. To assess their suitability for C–S–H, we selected MACE‐MP‐0 as a representative example [46, 47, 48]. Our results show that the performance of MACE‐MP‐0 is overall inferior to both csh_cp and our CSH‐MLP in describing tobermorite systems, underscoring that element coverage alone is insufficient for accurately resolving configurational diversity, particularly in complex, disordered systems such as C–S–H. For the C–S–H benchmark model, both MACE and csh_cp yield significantly poorer accuracy compared to CSH‐MLP. This difference is consistent with the fact that MACE‐MP‐0 and csh_cp are trained predominantly on crystalline configurations, with C–S–H‐related structures either absent or only minimally represented. Consequently, both models show lower predictive accuracy for the nanocrystalline C–S–H configuration examined here.

More fundamentally, these limitations reflect the underlying deficiencies of the dataset. From the perspective of dataset‐generation strategies, the training datasets of the MACE‐MP‐0 and csh_cp are typically biased toward nearly perfect crystal structures. This is reflected in the observation that MACE‐MP‐0 and csh_cp perform better on tobermorite crystals than on nanocrystalline C–S–H. Even when configurations from equilibrated MD trajectories are included in the training dataset, these remain near equilibrium and exclude non‐equilibrium regions, which are essential for stabilizing systems during MD simulations. These findings not only highlight the critical limitations of MACE‐MP‐0 when applied to C–S–H, but also underscore the advantages of our comprehensive C–S–H dataset and the resulting CSH‐MLP in addressing the unique challenges of this complex material.

The robustness of CSH‐MLP in predicting the C–S–H structure was further validated using two representative large‐scale models, bulk C–S–H and C–S–H gel, and compared against csh_cp and ReaxFF. Importantly, these benchmark models were constructed using methods different from those employed in this work, ensuring fairness by avoiding any artificial bias toward CSH‐MLP. Moreover, the use of widely accepted models enhances both the representativeness and credibility of the comparisons. As shown in Figure S8, both CSH‐MLP and ReaxFF successfully stabilize the C–S–H models throughout the simulations, whereas csh_cp produces spurious oxygen clustering due to inaccurate atomic interactions, ultimately leading to simulation failure. These results demonstrate that CSH‐MLP exhibits strong generalizability across diverse C–S–H structures and offers greater robustness than previously developed cement‐related MLPs.

The accuracy of CSH‐MLP in capturing interatomic interactions within the C–S–H model was examined by comparing the pair distribution function (PDF) and radial distribution function (RDF) predicted by CSH‐MLP with those obtained from ab initio molecular dynamics (AIMD) simulations and experimental measurements (Figure 5). In Figure 5a, the PDF predicted by CSH‐MLP closely aligns with AIMD results, accurately capturing the positions of the first two peaks: the Si─O peak at ∼1.65 Å and the Ca─O peak at ∼2.40 Å, both in good agreement with experimental measurements. The third peak, corresponding to the O─O interaction at ∼2.67 Å, begins to show a slight deviation from the experimental data. At longer ranges, additional minor discrepancies are observed between the calculated and experimental PDFs. These may arise from inherent limitations of the idealized tobermorite‐based C–S–H structural model, which does not incorporate structural defects or fully capture the complexity of the disordered nanostructure. Moreover, interference from impurities such as residual calcium hydroxide in the experimental samples could contribute to the observed deviations. Experimental uncertainties, including background subtraction errors and instrumental resolution limits, may also play a role.

**FIGURE 5:**
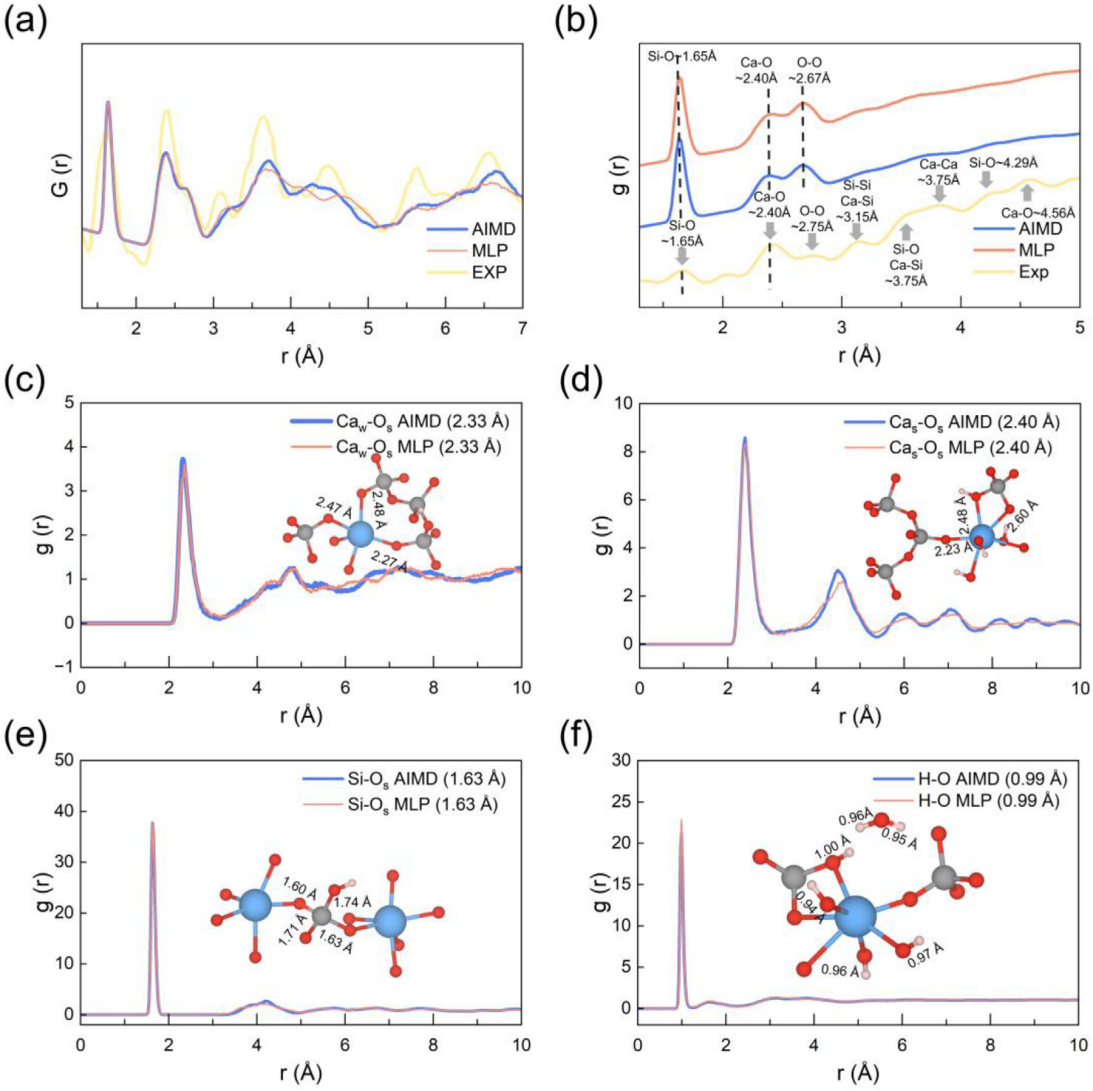
Structural analysis of the C–S–H model with Ca/Si = 1.5. (a) Pair distribution function (PDF) compared among CSH‐MLP, ab initio molecular dynamics (AIMD), and experimental data from C.E. White et al. [49]. (b) Radial distribution function (RDF) compared with experimental measurements from Soyer‐Uzun et al. [50]. (c–f) RDF comparisons between CSH‐MLP and AIMD for (c) Ca_W_‐O_S_, (d) Ca_S_‐O_S_, (e) Si‐O_S_, and (f) O─H atomic pairs. Values in parentheses indicate the positions of the first RDF peaks. O_S_ denotes silicate tetrahedral oxygen, Ca_W_ and Ca_S_ indicate interlayer and intralayer calcium, respectively, while Si, H, and O denote silicon, hydrogen, and oxygen atoms.

Figure 5b further compares RDFs derived from all‐atom simulations. The first three peaks are clearly resolved, demonstrating that the CSH‐MLP effectively captures the short‐range structural ordering. Notably, the RDF calculated by the CSH‐MLP exhibits excellent agreement with independent experimental data from two separate research groups [49, 50]. This consistency highlights the robustness and reliability of the CSH‐MLP in predicting short‐range atomic structures.

Bond distances between oxygen atoms from silicate tetrahedra (O_s_) and interlayer calcium atoms (Ca_w_), intralayer calcium atoms (Ca_s_) and silicon (Si), as well as between hydrogen (H) and oxygen (O) were analyzed through individual RDFs (Figure 5c–f). The close agreement between CSH‐MLP and AIMD simulations results confirms that the CSH‐MLP reliably reproduces the fundamental structural characteristics of the C–S–H models.

We further investigated the coordination environment of Ca in confined C–S–H spaces using CSH‐MLP. As shown in Table S3, increasing the confinement size from ∼8 to ∼29 Å leads to only a modest increase in the mean Ca coordination number. As the pore widens, the water content increases; correspondingly, the fraction of five‐coordinated Ca decreases, while the combined fraction of six‐ to eight‐coordinated Ca increase. This increasing trend in Ca coordination number with increasing pore size is consistent with prior MD simulations [51]. We note, however, that quantitative differences remain relative to earlier results obtained from implicit C–S–H surface models that represents C–S–H surfaces with smooth, uniformly charged walls. This simplification leads to a significantly higher Ca coordination number, particularly with fully hydrated Ca (i.e., Ca surrounded by an eight‐water hydration shell) becoming dominant. In contrast, CSH‐MLP enables more realistic C–S–H confinement environments in which a significant fraction of Ca remains surface‐anchored. As the pore expands, the number of Ca ions with complete hydration shells increases, which alleviates dense Ca packing at the surface and leads to a moderate increase in the overall mean coordination number.

### 2.5 Benchmarking of C–S–H Dynamic Properties

For benchmarking dynamic properties, we calculated the mean squared displacement (MSD) of different atomic species under ClayFF, ReaxFF, csh_cp, and CSH‐MLP for the DFT‐optimized benchmark C–S–H model (Figure 6a–d). Using the MSD analysis as a metric to quantify the deviation from the DFT‐optimized structure, the results show that, compared with ClayFF, ReaxFF yields lower MSD values, indicating that the relaxed structures predicted by ReaxFF remain closer to the DFT reference. Both CSH‐MLP and csh_cp yield extremely small and stable MSD values, indicating that the structures remain close to the initial DFT‐optimized configuration during the simulations. Notably, the MSD fluctuations of CSH‐MLP are more consistent and stable than those of csh_cp, suggesting weaker thermal vibrations of C–S–H under the CSH‐MLP potential. These results demonstrate that CSH‐MLP attains higher accuracy and stability than previously developed cement‐based MLPs.

**FIGURE 6:**
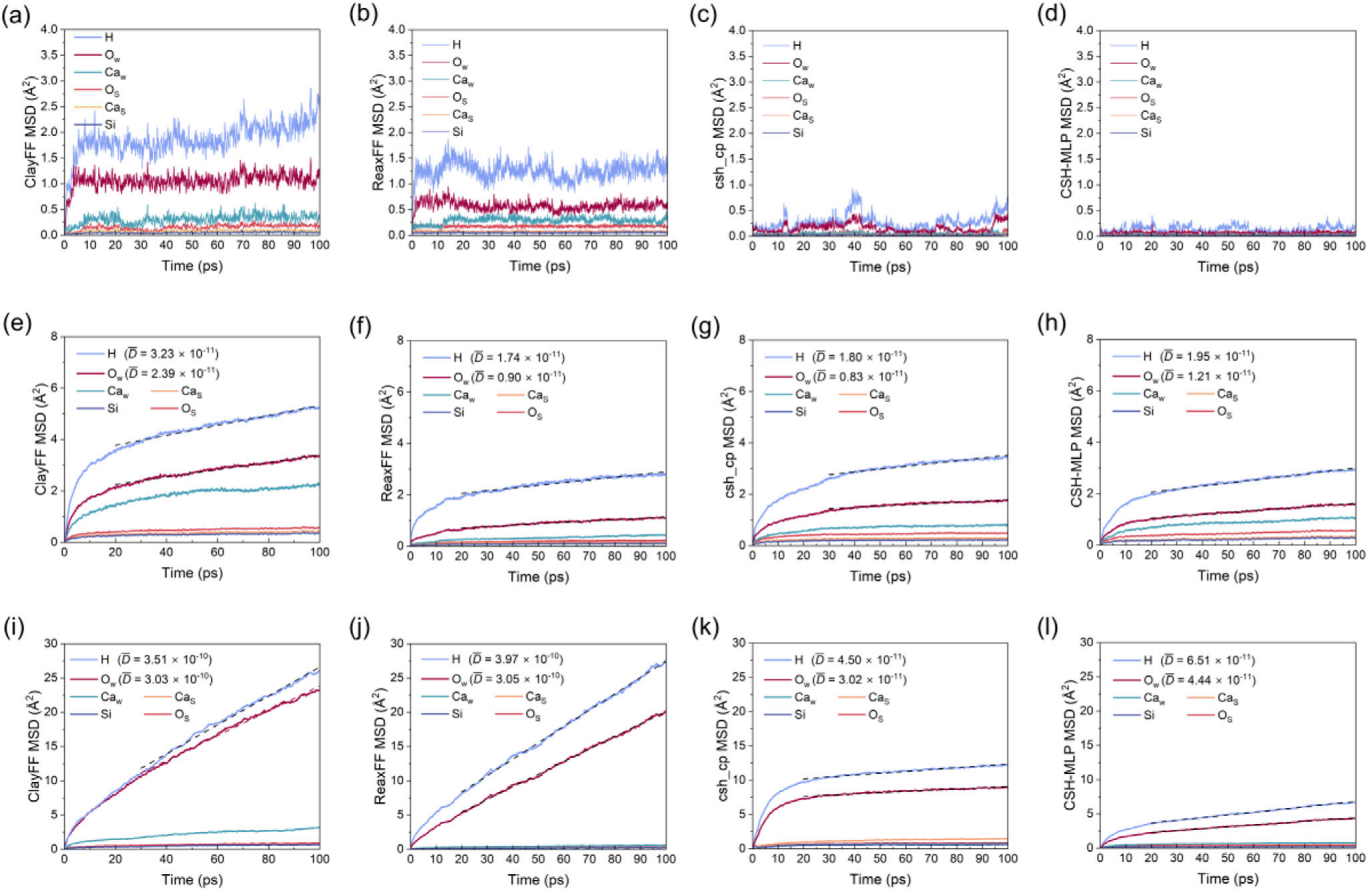
Mean square displacement (MSD) comparisons across force fields for three C–S–H systems. (a–d) MSDs for the DFT‐optimized benchmark C–S–H model using (a) ClayFF, (b) ReaxFF, (c) csh_cp, and (d) CSH‐MLP. (e–h) MSD for the large‐scale bulk C–S–H model (8456 atoms) using (e) ClayFF, (f) ReaxFF, (g) csh_cp, and (h) CSH‐MLP. (i–l) MSD for the C–S–H gel model (10 109 atoms) using (i) ClayFF, (j) ReaxFF, (k) csh_cp, and (l) CSH‐MLP. In all panels, the black dashed line indicates the diffusion stage used for calculating diffusion coefficients, which are expressed in the unit of m^2^·s^−1^ and reported in parentheses. O_S_ denotes silicate tetrahedral oxygen, O_W_ denotes interlayer water oxygen, Ca_W_ and Ca_S_ indicate interlayer and intralayer calcium, respectively, and Si and H denote silicon and hydrogen.

Validating the dynamic performance of CSH‐MLP on large‐scale bulk C–S–H (8456 atoms) and C–S–H gel (10 109 atoms) models is crucial to ensure its capability for stably simulating disordered C–S–H (Figure 6e–l). For both systems, the MSD of O and H atoms predicted by ClayFF exhibits a rapid, unphysical increase, which is inconsistent with the expected confinement of water and hydroxyl groups by the C–S–H surface or the ∼1 nm nanopore. ReaxFF yields reasonable MSD results for the bulk model, consistent with Li et al. [18], but also shows spurious increases for O and H in the gel system. Both csh_cp and CSH‐MLP predict similarly accurate MSD values for O and H atoms across bulk and gel systems. However, trajectory analysis reveals that the C–S–H gel simulated with csh_cp undergoes severe structural artifacts, including extensive atomic clustering and the formation of vacuum regions within the water layer (Movies S1–S2 and Figure S8). In contrast, no structural artifacts were observed in the simulations using ClayFF, ReaxFF, or CSH‐MLP (Movies S3–S5). These results confirm that csh_cp fails to equilibrate gel structures, whereas CSH‐MLP achieves a level of stability, accuracy, and applicability that clearly surpasses previously developed MLPs.

We further calculated the diffusion coefficients of water molecules from the MSD in large‐scale C–S–H models. Compared with the values obtained using ClayFF (3.03 × 10^−10^ m^2^/s) and ReaxFF (3.05 × 10^−10^ m^2^/s), the diffusion coefficient predicted by CSH‐MLP (4.44 × 10^−11^ m^2^/s) is closer to the range reported in previous studies (6.00–15.00 × 10^−11^ m^2^/s) [52, 53, 54]. This demonstrates that, despite being developed solely on bulk C–S–H building block models, it remains applicable for studying the confined diffusion of water in C–S–H nanopores. Taken together, these dynamic tests confirm that CSH‐MLP proves effective for investigating surface‐related phenomena in complex C–S–H systems.

Beyond water transport, we further assessed the ability of CSH‐MLP to describe local chemical dynamics involving water and hydroxyl species. For a representative C–S–H model with Ca/Si = 1.3, CSH‐MLP closely reproduces the AIMD evolution of H_2_O/Si and OH/Si over 10 ps, whereas ReaxFF exhibits substantially larger deviations (Figure S7). This agreement demonstrates an improved description of local water and hydroxyl rearrangements, supporting the applicability of CSH‐MLP to water‐mediated processes at C–S–H interfaces.

### 2.6 Benchmarking of C–S–H Mechanical Properties

Figure 7a,b reports the elasticity tensor of the C–S–H benchmark model predicted by CSH‐MLP and csh_cp together with their deviations from DFT. White indicates minimal deviation, blue indicates underestimation, and orange indicates overestimation. Compared with the previously developed csh_cp, CSH‐MLP shows greater agreement with DFT. It should be noted that, for empirical force fields, calculations on small models tend to amplify intrinsic errors. For instance, in ClayFF, the strong covalent character of Si─O bonds and the ionic nature of Ca─O bonds are oversimplified by representing both interactions with Lennard‐Jones potentials. In principle, this should lead to an underestimation of C_22_ (C_ij_, where the indices i and j represent the stress and strain components, respectively) due to this direction is dominated by Si─O bonds, yet the value is instead overestimated, as shown in Figure S9a.

**FIGURE 7:**
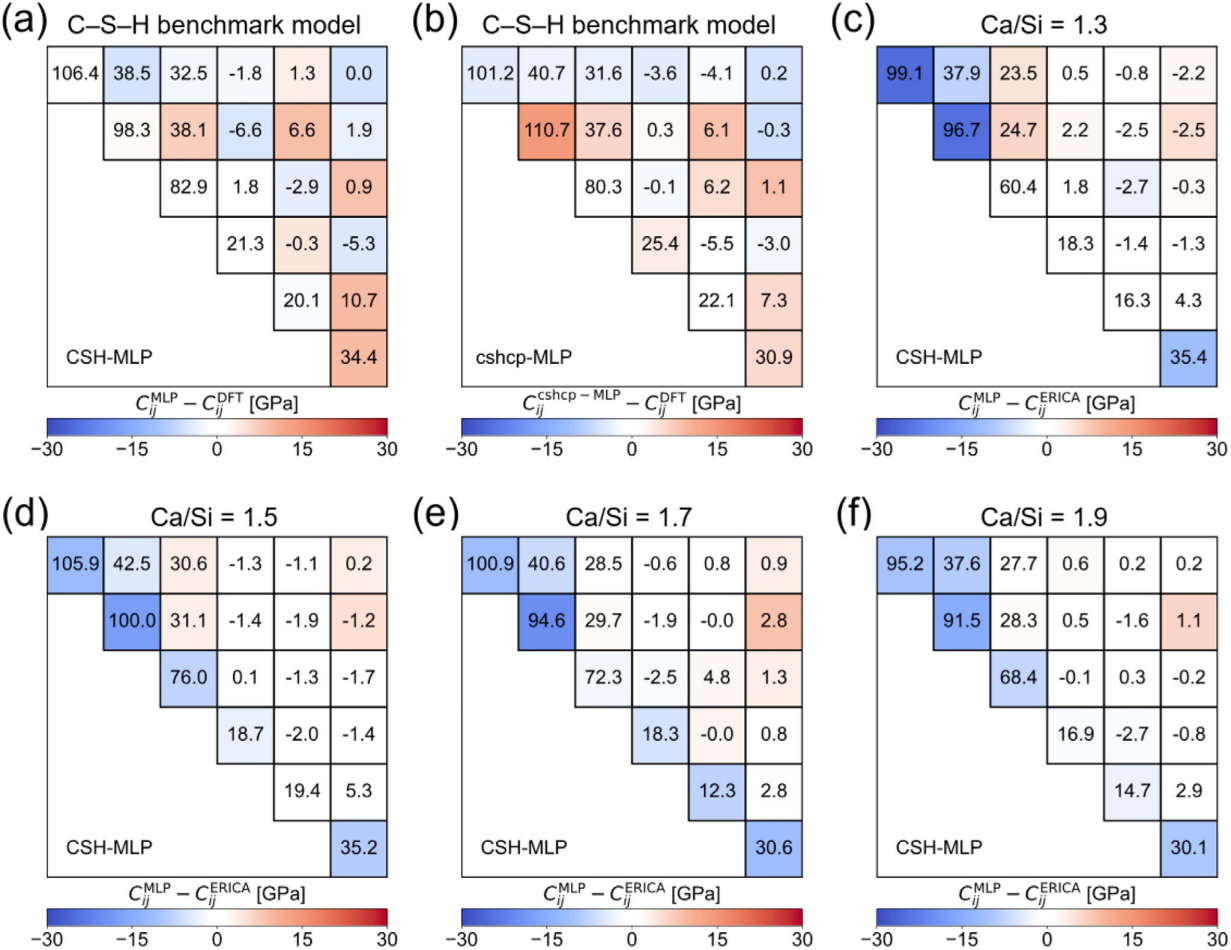
Elasticity tensor of the C–S–H benchmark model and large‐scale C–S–H models, along with their comparisons across different computational methods. Panels (a,b) present the elastic constants of the C–S–H benchmark model predicted by the machine learning potential proposed in this work (CSH‐MLP), the csh_cp machine learning potential, respectively, together with their deviations from the DFT results. Panels (c–f) display the elastic constants of large‐scale C–S–H models with Ca/Si ratios of 1.3, 1.5, 1.7, and 1.9, respectively, as predicted by the MLP. The differences relative to the ERICA FF predictions are used to evaluate the composition‐dependent accuracy of the MLP.

Figure 7c–f shows the elasticity tensor of large bulk C–S–H models with Ca/Si ratios from 1.3 to 1.9 predicted by CSH‐MLP, with their deviations from ERICA FF. The predominantly blue differences indicate that ERICA FF systematically yields higher values than CSH‐MLP, consistent with the benchmark‐model tests. ERICA FF's overestimation is larger for C_11_ and C_22_ than for C_33_, especially for C_22_. Since C_22_ primarily reflects stiffness along the silicate‐chain direction and C_11_ captures the Ca─Si layer network strength between chains, this pattern suggests that the empirical force field misrepresents the strength of the Ca─Si layer and the silicate chains. The discrepancy likely arises from the less accurate description of the strong covalent Si─O bonding embedded in ERICA FF. Detailed numerical values of the elastic constants are provided in Table S2.

Analysis of the normal components of the elasticity tensor reveals a clear non‐monotonic trend in the mechanical response of C–S–H. As Ca/Si increases from 1.3 to 1.5, C_11_, C_22_ and C_33_ grow from 99.12 to 105.94 GPa, 96.71 to 100.02 GPa, and 60.39 to 76.04 GPa respectively; with further increases in Ca/Si, they drop to 95.15, 91.46, and 68.44 GPa, respectively. At lower Ca/Si, added Ca densifies the interlayer and strengthens interlayer cohesion, so stiffness increases while silicate chains remain largely continuous. At higher Ca/Si, however, progressive silicate‐chain breakage together with excessive Ca enrichment in the interlayer introduces disorder and reduces effective density, thereby lowering stiffness in all directions. These trends reconcile the apparent discrepancy between HP‐XRD observations of monotonically increasing measured strength with Ca/Si [55, 56], and the conventional expectation that higher Ca/Si promotes disorder and softening in C–S–H [57, 58]. Our simulations provide an atomic‐scale explanation for this behavior, linking the experimental observations to the structural evolution of C–S–H.

The bulk modulus, shear modulus, Young's modulus, and Poisson's ratio of C–S–H were first evaluated on the benchmark model using both CSH‐MLP and the previously developed MLP, csh_cp, with results directly compared to DFT calculations. As shown in Table 2, CSH‐MLP achieves excellent agreement with DFT, reproducing all four mechanical properties with deviations of ∼1%. This accuracy is largely attributed to its precise prediction of the dominant diagonal components of the elastic tensor (Figure 7a,b). By contrast, csh_cp shows clear discrepancies, including overestimations of the shear and Young's moduli by 7.5% and 6.65%, respectively, and an underestimation of Poisson's ratio by −3.33%.

**TABLE 2:** Benchmarking of CSH‐MLP and csh_cp against DFT for mechanical properties, including the Hill‐averaged bulk modulus, shear modulus, Young's modulus and Poisson's ratio. Comparative analyses are presented across CSH‐MLP, csh_cp, ReaxFF, ClayFF, and ERICA FF on the large C–S–H models with varying Ca/Si ratios. The benchmark C–S–H model is a 2 × 2 × 1 supercell with a Ca/Si ratio of 1.65, while the large C–S–H models are 4 × 4 × 2 supercells with varying Ca/Si ratios. Values in parentheses denote the percentage deviations from DFT (for the C–S–H benchmark model) and from CSH‐MLP (for the large C–S–H models), respectively.

Model	Method	Bulk modulus (GPa)	Shear modulus (GPa)	Young's modulus (GPa)	Poisson's ratio
C–S–H benchmark model	DFT	55.19	24.40	63.80	0.31
	CSH‐MLP	55.77	24.66	64.49	0.31
		(1.04%)	(1.07%)	(1.08%)	(0.09%)
	csh_cp	55.89	26.23	68.04	0.30
		(1.27%)	(7.50%)	(6.65%)	(−3.33%)
Large C–S–H model (Ca/Si = 1.3)	CSH‐MLP	45.57	23.88	60.98	0.28
	csh_cp	44.62	24.07	61.20	0.27
		(−2.09%)	(0.78%)	(0.36%)	(−2.00%)
	ReaxFF	52.34	26.19	67.34	0.29
		(14.87%)	(9.67%)	(10.42%)	(3.1%)
	ClayFF	37.64	20.27	51.56	0.27
		(−17.41%)	(−15.11%)	(−15.45%)	(−1.91%)
	ERICA FF	47.75	28.45	71.20	0.25
		(4.79%)	(19.13%)	(16.76%)	(−9.19%)
Large C–S–H model (Ca/Si = 1.5)	CSH‐MLP	53.47	25.34	65.65	0.30
	csh_cp	51.87	25.13	64.90	0.29
		(−2.99%)	(−0.84%)	(−1.14%)	(−1.32%)
	ReaxFF	49.12	24.72	63.50	0.28
		(−8.13%)	(−2.47%)	(−3.28%)	(−3.66%)
	ClayFF	40.73	19.61	50.69	0.29
		(−23.84%)	(−22.62%)	(−22.79%)	(−0.95%)
	ERICA FF	56.54	29.82	76.08	0.28
		(5.74%)	(17.66%)	(15.88%)	(−6.64%)
Large C–S–H model (Ca/Si = 1.7)	CSH‐MLP	50.43	21.85	57.27	0.31
	csh_cp	53.54	25.19	65.32	0.30
		(6.16%)	(15.28%)	(14.06%)	(−4.53%)
	ReaxFF	43.20	22.32	57.12	0.28
		(−14.34%)	(2.15%)	(−0.26%)	(−10.01%)
	ClayFF	41.60	19.06	49.61	0.30
		(−17.50%)	(−12.77%)	(−13.38%)	(−3.04%)
	ERICA FF	54.16	29.00	73.82	0.27
		(7.40%)	(32.71%)	(28.90%)	(−12.19%)
Large C–S–H model (Ca/Si = 1.9)	CSH‐MLP	48.29	21.85	56.96	0.30
	csh_cp	53.58	23.06	60.51	0.31
		(10.95%)	(5.55%)	(6.23%)	(2.75%)
	ReaxFF	44.37	20.85	54.08	0.30
		(−8.12%)	(−4.58%)	(−5.06%)	(−2.16%)
	ClayFF	38.12	16.54	43.36	0.31
		(−21.06%)	(−24.29%)	(−23.88%)	(2.31%)
	ERICA FF	52.72	26.26	67.57	0.29
		(9.18%)	(20.19%)	(18.62%)	(−5.60%)

Following the benchmarking, we further applied CSH‐MLP to calculate the mechanical properties of large, defect‐rich C–S–H models (4×4×2 supercells) spanning Ca/Si ratios from 1.3 to 1.9 (Table 2). These larger models better capture the intrinsic heterogeneity of C–S–H, where composition and defect structures vary spatially, and thus provide a more realistic representation of its nanostructure and mechanical response. Unlike the benchmark model, which can be directly validated against DFT due to its relatively small size, DFT benchmarking of such large‐scale models is computationally infeasible. Therefore, comparisons of the calculated mechanical properties using CSH‐MLP and other established force fields (ClayFF, ReaxFF, ERICA FF, and csh_cp) were conducted to evaluate their relative performance and robustness.

The results demonstrate that ClayFF and ReaxFF generally predict lower mechanical properties than CSH‐MLP. ERICA FF, as the most accurate empirical force field tailored for cementitious materials, shows only slight overestimations relative to CSH‐MLP. For csh_cp, the predictions are consistent with CSH‐MLP at lower Ca/Si ratios (1.3 and 1.5), where the structures are relatively ordered, and silicate chains remain largely intact. This agreement arises because the testing models share structural similarities with the csh_cp training dataset, which is dominated by tobermorite‐like configurations. However, at higher Ca/Si ratios (1.7 and 1.9), where structural disorder becomes more pronounced, these similarities diminish. As a result, csh_cp shows significant deviations, particularly in shear modulus (15.28%) and Young's modulus (14.06%). These results highlight the necessity of constructing datasets that span a broad configurational space to ensure the accuracy and generalizability of an MLP. Notably, the bulk modulus predicted by CSH‐MLP at Ca/Si = 1.3 shows excellent agreement with the experimental value of 45.6 GPa from high‐pressure X‐ray diffraction (HP‐XRD) measurements [59], supporting the accuracy of this specific prediction.

As the Ca/Si ratio increases, the bulk modulus rises from 45.57 GPa (Ca/Si = 1.3) to a peak of 53.47 GPa (Ca/Si = 1.5), and then declines to 48.29 GPa (Ca/Si = 1.9). The increasing trend below Ca/Si = 1.5 is consistent with HP‐XRD results [55, 56], and can be attributed to structural densification, as the C–S–H structure retains tobermorite‐like order at lower Ca/Si ratios [60]. The decrease above Ca/Si = 1.5 differs from the monotonic increase suggested by the available HP‐XRD data but is consistent with increasing structural disorder and mechanical softening at higher Ca/Si ratios [57, 58]. Ensemble studies based on 150 independent structures by Qomi et al. [57] and ten structures per composition in our recent work [61] reported similar composition‐dependent mechanical responses, including a change near Ca/Si ≈ 1.5. These results support the physical plausibility of the trend observed here.

Integrating the trends of the normal elasticity coefficients with the Ca/Si ratio, we contend that the two prevailing interpretations are not contradictory but complementary. HP‐XRD studies emphasize interlayer densification, give limited consideration to silicate‐chain evolution, and offer sparse data for Ca/Si > 1.3; The previous simulation viewpoint focuses on defect formation (e.g., reduced mean chain length, MCL), but it is constrained by force‐field fidelity. We reconcile these perspectives as follows: at lower Ca/Si (≈≤1.5), interlayer densification dominates, raising the bulk modulus despite incipient chain scission; at higher Ca/Si, decreasing interlayer density together with progressive chain fragmentation leads to a decline in the bulk modulus.

### 2.7 Computational Speed Comparison

To evaluate its computational performance, we compared the simulation speeds of CSH‐MLP, ReaxFF, and csh_cp across systems of varying sizes. As shown in Figure 8, CSH‐MLP consistently outperforms ReaxFF in computational efficiency under identical hardware and simulation setups. With a conservative timestep of 0.2 fs (the maximum stable timestep for ReaxFF), CSH‐MLP runs 3.5–6.0× faster across system sizes. Owing to its superior numerical stability, CSH‐MLP can be advanced with timesteps up to 1.0 fs without loss of stability, which increases its effective efficiency advantage to 16.8–29.9× over ReaxFF. Compared with csh_cp, CSH‐MLP further improves performance, delivering on average ∼1.4× faster simulation speeds across different system sizes. In addition, CSH‐MLP exhibits faster pressure relaxation and reaches a stable pressure regime more rapidly than ERICA FF, as shown in Figure S4. Furthermore, we also compared the computational efficiency of the MACE‐MP‐0, a general‐purpose MLP, with CSH‐MLP and csh_cp. The simulation speed of MACE‐MP‐0 for C–S–H (the C–S–H benchmark model containing 180 atoms) on a single RTX 4090 is only 0.8 ns/day, compared to 21.8 ns/day for CSH‐MLP and 14.0 ns/day for csh_cp under identical conditions, demonstrating that CSH‐MLP is more computationally efficient than MACE‐MP‐0 in simulating C–S–H. These results underscore the superior scalability and practical potential of CSH‐MLP for large‐scale simulations of cementitious materials.

**FIGURE 8:**
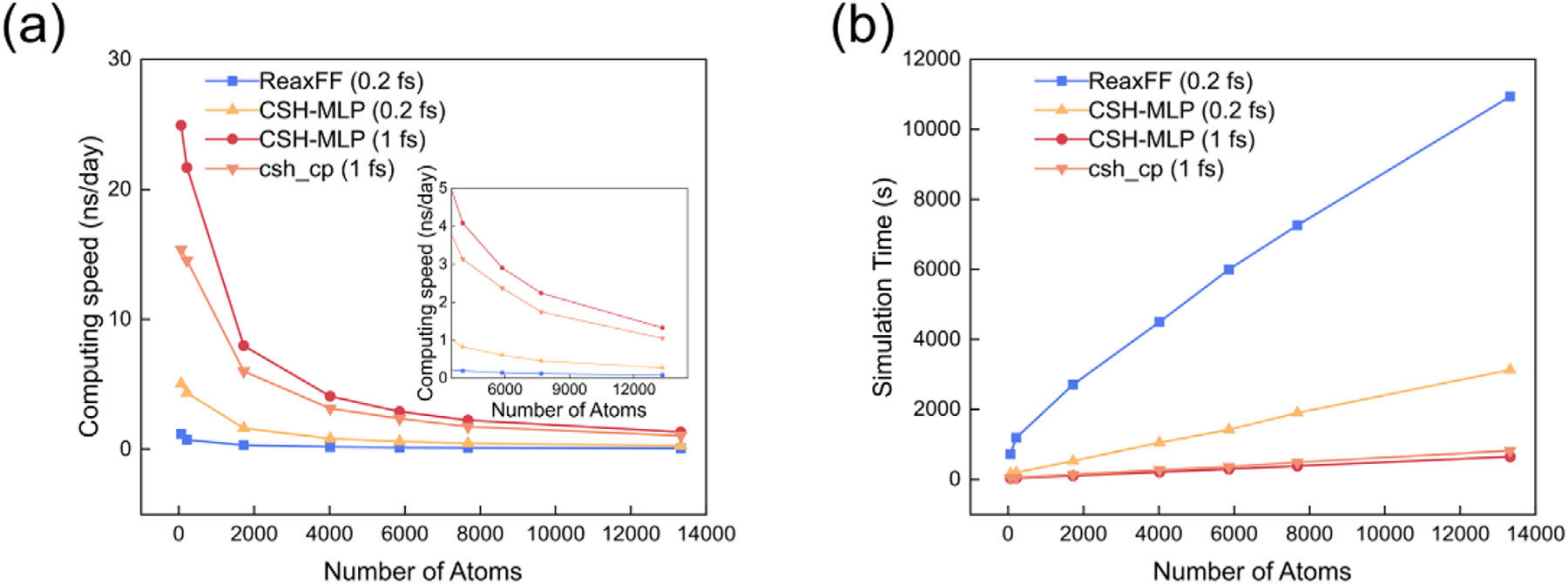
Computational performance comparison of CSH‐MLP, ReaxFF, and csh_CP. (a) Simulation speeds as a function of system size (number of atoms). (b) Total time required to perform a 10 ps canonical ensemble (NVT) simulation. All simulations were carried out under identical conditions on a single NVIDIA RTX 4090 GPU.

## 3 Discussion

One of the central motivations of this study is to develop a force field that can extend the QM accuracy to large systems and long simulations that were previously infeasible. Though providing two demonstrations by applying CSH‐MLP to large‐scale bulk C–S–H and C–S–H nanopore models from the literature, we achieve the atomistic simulations of C–S–H with more than 10 000 atoms under the DFT‐level accuracy for nanosecond time scales, especially using the models that were not part of our training dataset. Furthermore, CSH‐MLP shows higher computational efficiency than ReaxFF across different atomic scales, while maintaining accuracy in predicting structural, dynamic, and mechanical properties. This combination of accuracy, scalability, and efficiency establishes CSH‐MLP as a foundational tool for atomistic simulations of cementitious materials, enabling large‐scale C–S–H simulations with near‐DFT accuracy at substantially reduced computational cost.

Another central motivation of this work is to establish an initial atomistic structure database for cement, focusing on the most important and complex hydration product in cementitious materials, C–S–H. In contrast to previous datasets limited to tobermorite and C–S–H with specific compositions [18], the present work constructs of a C–S–H dataset spans nearly 700 distinct C–S–H compositions and more than 50 000 DFT‐labeled configurations, covering broad variations in Ca/Si ratios, hydration states and defect topologies systems, we present the largest and the most comprehensive, representative, and openly accessible DFT‐labeled C–S–H dataset to date, which is the first step toward the construction of the ‘cement genome’. By characterizing the structural features of 1890 C–S–H models sampled from these ∼700 compositions using the SOAP fingerprinting method, we found that, although all are classified as C–S–H, their atomic arrangements and defect distributions exhibit significant differences. This further underscores that a high‐accuracy, stoichiometry‐generalizable MLP for C–S–H must be trained on a broad and representative dataset that fully captures diverse defect types.

The dataset‐generation methodology and modeling framework proposed here provide a robust foundation that can be systematically expanded to other cementitious phases, while the CSH‐MLP developed in this study serves as an efficient and accurate tool for generating large‐scale, high‐fidelity data. This framework will directly support and accelerate the construction of a comprehensive “cement genome” in a data‐driven manner. Once the “cement genome” has been constructed, we will enable to develop a general‐purpose MLP in cement science, which can be transferable across crystalline, nanocrystalline, hydrous, and anhydrous cementitious systems. Ultimately, by accurately capturing the structure‐property relationships of cementitious materials at the atomic scale, we hope these insights can instruct the design and preparation of materials at the macroscopic.

While the present work establishes a comprehensive dataset and demonstrates the strong generalizability of CSH‐MLP across diverse C–S–H systems as well as its high accuracy in calculating structural, dynamics and mechanical properties, several limitations remain. First, the use of ERICA FF for trajectory sampling may introduce some sampling bias, as configurational regions not readily accessible on its potential‐energy surface could remain underrepresented. Second, all DFT labels were generated using the PBE functional with Grimme D3 dispersion correction to improve the description of weak interactions. However, the present MLP does not include an explicit long‐range correction. For applications where long‐range interactions are critical, the dataset should be retrained using an MLP framework with explicit long‐range treatment. Third, additional dataset expansion and refinement will be needed to address downstream tasks involving more complex chemistries, such as models with elements beyond Ca, Si, O, and H (e.g., carbonation, sulfate attack, alkali and minor‐element uptake (e.g., Na, K, and Al), and organic/inorganic interactions) or systems that deviate significantly from the C–S–H framework (e.g., nucleation processes and solid/liquid interfaces). Nevertheless, the C–S–H dataset developed in this work provides a critical foundation for such downstream extensions. With only modest amounts of system‐specific DFT‐labeled data, CSH‐MLP can be fine‐tuned to address these broader challenges. Finally, CSH‐MLP can in principle describe reactive events involving bond breaking or formation. However, such processes were not explicitly sampled or targeted during dataset construction. Its applicability to specific reactive processes therefore requires further validation.

In summary, this work presents a generalizable and robust MLP for C–S–H, CSH‐MLP, capable of achieving near–QM accuracy. To develop CSH‐MLP, we propose a systematic data‐generation and evaluation framework designed to ensure dataset diversity, broad chemical‐space coverage, and representativeness across C–S–H compositions and local environments. Using this strategy, we construct a compositionally diverse and openly accessible DFT‐labeled C–S–H dataset spanning approximately 700 compositions and more than 50 000 configurations. By visualizing the structural features of the dataset and evaluating the maximal standard deviation of atomic forces for C–S–H models both within and beyond the training set, we demonstrate that CSH‐MLP is broadly applicable to a variety of C–S–H structures, including those generated by different construction methods and tobermorite phases. CSH‐MLP closely reproduces DFT predictions of structural, dynamic, and mechanical properties while offering greater numerical stability than ReaxFF and achieving a 3.5–6.0× speedup over ReaxFF at the same integration time step and an effective 16.8–29.9× speedup with a larger stable time step. Furthermore, CSH‐MLP offers new insights into the nanoscale mechanics of C–S–H, resolving long‐standing discrepancies between atomistic simulations and experimental high‐pressure X‐ray diffraction data. By enabling accurate, large‐scale, and long‐timescale simulations of cementitious systems, this work offers a practical and scalable strategy for integrating cementitious materials into the broader Materials Genome Initiative. Our work advances atomistic simulations of cementitious materials and opens new avenues for the bottom‐up design of sustainable cement systems.

## 4 Methods

### 4.1 Construction of C–S–H Atomic Structures

The C–S–H atomic structures used in this study were generated using the pyCSH program [34], which constructs C–S–H models based on the building block methodology [10] and the structural framework derived from 14 Å tobermorite [43] with high efficiency. By setting the calcium‐to‐silicon (Ca/Si) ratio precision constraint to 0.9 and selecting the independent mode in pyCSH, the program can exhaustively generate all plausible C–S–H models spanning Ca/Si ratios from 0.83 to 2.5. After structural optimization, the lattice parameters of the bulk C–S–H models fall within the ranges of a = 12.70–13.88 Å, b = 14.30–15.56 Å, and c = 11.05–19.13 Å. To enhance the stability of the machine learning potential (MLP) for the C–S–H system, perturbed C–S–H models were generated with perturbation magnitudes of 2%, 5%, 10%, 15%, and 20%. To evaluate the applicability of the developed MLP, we also examined additional C–S–H structures that were not included in the training or test datasets. These include a C–S–H model generated using the Pellenq method with a Ca/Si ratio of 1.7 [8], and another incorporating a 1 nm nanopore filled with water [44].

### 4.2 Atomistic Simulation Setup

To generate reliable C–S–H models and a diverse set of training data, a rigorous structural optimization strategy was adopted in this study. Three independent molecular dynamics (MD) simulations of C–S–H were initially performed in the canonical (NVT) ensemble at 298.15 K for 50 000 steps with a timestep of 0.05fs, this simulation progressed in stages: first allowing only water molecules to move, then including hydroxyl groups and hydroxide ions, and finally permitting the motion of all calcium atoms. Energy minimization of the simulation box was then carried out to optimize the atomic arrangement. The heating – quenching MD process lasted for 1.65 ns with a timestep of 0.2 fs. Final equilibration was conducted under the isothermal–isobaric (NPT) ensemble with a Nosé‐Hoover thermostat at 298.15 K and 1 atm for 800 ps with a timestep of 0.2fs. The production run was performed for 1 ns within the NPT ensemble at a temperature of 298.15 K and a pressure of 1 atm with a timestep of 0.5 fs given the high efficiency of the rigorous minimization procedure. ERICA FF force field with a polarizable core–shell model [62] was used in [20]. All simulations were performed by the LAMMPS code [63].

Density functional theory (DFT) based geometry optimizations and single‐point energy calculations were implemented in the CP2K/Quickstep code [64]. The generalized gradient approximation (GGA) with the Perdew–Burke–Ernzerhof (PBE) exchange–correlation functional was employed, along with Grimme's D3 dispersion correction to account for van der Waals interactions [65, 66]. Core–valence interactions were described using Goedecker‐Teter‐Hutter (GTH) pseudopotentials [67, 68] within the Gaussian and plane‐wave (GPW) framework [69]. Wavefunctions were expanded using a double‐ζ valence polarized (DZVP) basis set and an auxiliary plane‐wave basis set with a cutoff energy of 400 Ry [70, 71]. The Brillouin zone was sampled using the Γ‐point approximation. The convergence thresholds for self‐consistent field iterations were set to 10^−5^ for geometry optimization and 10^−6^ for single‐point energy calculations. Wave functions were converged to 10^−^^10^ and 10^−^^12^, respectively.

### 4.3 Structural Analysis of C–S–H Dataset

Smooth Overlap of Atomic Positions (SOAP) [35, 72] was used to describe the structural property of the C–S–H dataset. SOAP captures the local atomic configurations through atomic coordinates, effectively representing the spatial relationships within the system. SOAP is implemented in the DScribe Python library, which encodes atomic neighborhoods by performing a Gaussian‐smeared atomic density expansion [72]. This expansion utilizes orthogonal basis functions, comprising spherical harmonics and radial basis functions, to represent the spatial arrangement of atoms. The resulting SOAP descriptors are averaged over atomic centers using an “inner”‐averaging scheme, yielding a high‐dimensional conformational fingerprint that characterizes the overall structure.

Principal component analysis (PCA) and t‐distributed stochastic neighbor embedding (t‐SNE) were employed to reduce the dimensionality of high‐dimensional data, enabling the visualization of spatial information within the structure. These techniques also facilitate the identification of correlations between specific features, such as the calcium‐silicate and water‐silicate ratios.

### 4.4 Training of the C–S–H Machine Learning Potential

The C–S–H machine learning potential (CSH‐MLP) was trained using the DeePMD‐kit package with the smooth edition [24, 25]. DeePMD‐kit requires atomic coordinates, energies, and forces, as well as simulation cell parameters, as training data. The architecture of the embedding and fitting networks consisted of layers with sizes (25, 50, 100) and (240, 240, 240), respectively [28]. A truncation radius of 6.0 Å was utilized to define the local atomic environment, and a cosine smoothing function was applied starting at 0.5 Å from the truncation point. The learning rate was initially set to 1.0 × 10^−3^ and decayed exponentially to 1.0 × 10^−8^ every 5000 training steps. The batch size was set as 1 during the training procedure. The values of ideal energy and force in the loss function during the optimization process ranged between 0.02 and 2, and 1000 and 1, respectively.

The dataset was divided into training and test sets with a 95:5 ratio using a group‐wise split. Configurations derived from the same parent structure or MD trajectory were assigned exclusively to either the training or test set, thereby avoiding overlap between structurally related configurations.

The final CSH‐MLP was trained for a total of 4.0 × 10^6^ steps, with each set of 100 steps requiring approximately 1.9 s on a single NVIDIA V100 GPU. After training, the CSH‐MLP was further compressed following the model compression scheme in reference [73] to accelerate the machine learning molecular dynamics (MLMD) while keeps almost the same accuracy.

### 4.5 Reliability Evaluation of CSH‐MLP During MLMD Simulations

The reliability of CSH‐MLP during MLMD simulations was assessed based on the maximal standard deviation of atomic forces predictions (ε_*F*_) from four independently trained MLPs. Specifically, MLMD simulations were performed using one of the MLPs, while the remaining three were used to predict atomic forces for configurations sampled at specific intervals along the trajectories. For each configuration, ε_*F*_ was computed as follow:

(1) $$\varepsilon_{F}=\max\sqrt{<{\left|\left|F_{i}-<F_{i}>\right|\right|}^{2}>}$$

where i is the i^th^ atom in the system, and <…> indicates the expectation with respect to the ensemble of the models and are estimated by the average of model predictions.

Four CSH‐MLPs were trained concurrently based on the same training dataset but with different initial weights, each trained for 8.0 × 10^5^ using the DP‐GEN package [74]. Five MLMD simulations were then performed using C–S–H models randomly selected across Ca/Si ratios ranging from 0.91 to 2.06. A comprehensive structural equilibration protocol, combining NPT and NVT ensembles with a heating‐quenching process, was employed to compute ε_*F*_. To further assess the applicability and robustness of the CSH‐MLP, two additional C–S–H models constructed using the widely adopted Pellenq scheme [8, 44], along with three tobermorite models of varying interlayer spacings: 9 Å [41], 11 Å [42], and 14 Å [43], were tested.

### 4.6 Structural Features and Mechanical Properties Calculations

Lattice constants were determined through initial energy minimization, followed by a 250 ps MD equilibration and a 750 ps production run. All simulations were performed using anisotropic NPT integration, allowing both cell dimensions and angles to vary throughout. The general‐purpose MLP benchmark used the publicly released MACE‐MP‐0 medium model developed by Batatia et al. [46, 47, 75], without system‐specific retraining, fine‐tuning, or additional corrections.

The atom pair distribution function (PDF) and radial distribution function (RDF) were computed by post‐processing the production trajectories from the final 10 ps ab initio molecular dynamics (AIMD) and final 0.5 ns MLMD. AIMD simulations employed a timestep of 1 fs, with hydrogen atoms assigned the deuterium mass to reduce high‐frequency O–H vibrations and improve numerical stability [76, 77, 78]. A canonical sampling approach using velocity rescaling with a time constant of 200 fs was applied to maintain the system temperature at 298.15 K. All other settings were consistent with those used in the DFT calculations. Hydrogen atoms were excluded from PDF analysis. The partial PDFs were combined using the multicomponent weighting scheme [79]. The PDF (*G*(*r*)) was calculated according to Equation (2):

(2) $$G\;\left(r\right)=\sum_{\alpha \beta }\frac{c_{\alpha }c_{\beta }b_{\alpha }b_{\beta }}{{<b>}^{2}}G_{\alpha \beta }\left(r\right)$$

where *b* represents the atomic scattering lengths, and *c* denotes the atomic fraction of species. The summation runs over all atomic species pairs in the system. *G*_αβ_(*r*) represents the partial pair distribution functions.

During the dynamic tests, when the initial model was the DFT‐optimized C–S–H model, 100 ps NVT calculations were performed using different force fields (ClayFF, ReaxFF, csh_cp, and CSH‐MLP) with a time step of 0.2 fs. When the initial model was one of the two stable large‐scale C–S–H models constructed by Duque‐Redondo et al. [44] using the Pellenq method [8], energy minimization was first performed with different force fields, followed by 100 ps NVT calculations. Except for ReaxFF, which used a 0.2 fs time step, ClayFF, csh_cp, and CSH‐MLP all used a 1 fs time step. The visualization movies were generated using OVITO [80]. The formula for calculating MSD(t) is given in (3):

(3) $$MSD\;\left(t\right)=\frac{1}{N}\sum_{i}^{N}{\left|r_{i}\left(t\right)-r_{i}\left(0\right)\right|}^{2}$$

DFT calculations of the mechanical properties of C–S–H were performed using the stress–strain method implemented in the VASP code [81]. Classical MD and MLMD calculations were carried out following energy minimization and box relaxation, using ClayFF, ReaxFF, ERICA FF, csh_cp, and CSH‐MLP. A timestep of 1 fs was used for all force fields. Each simulation was run for a total of 1.69 ns, comprising 13 deformation cycles. Each cycle consisted of 100 ps of equilibration followed by 30 ps of production. A uniaxial deformation amplitude of 2% was applied, and the resulting stress responses were used to compute the elastic stiffness tensor. Mechanical properties, including bulk modulus, shear modulus, Young's modulus and Poisson's ratio, were derived from the stiffness matrix using the Voigt‐Reuss‐Hill approximation [82, 83, 84]. The full results are summarized in Table S1.

## Author Contributions

Y.L. conceived and supervised the project. Y.L. and C.C. designed the research. Y.L. and C.C. performed the simulations and analyzed the results. Y.L., C.C., R.J.‐M.P., Z.L. and J.L. discussed and interpreted the results. Y.L. and C.C. wrote and revised the manuscript. All authors reviewed and approved the final version of the manuscript.

## Conflicts of Interest

The authors declare no conflicts of interest.

## Supporting information




**Supporting File 1**: advs77942‐sup‐0001‐SuppMat.docx

**Supporting File 2**: advs77942‐sup‐0002‐MovieS1.mov.

**Supporting File 3**: advs77942‐sup‐0003‐MovieS2.mov.

**Supporting File 4**: advs77942‐sup‐0004‐MovieS3.mov.

**Supporting File 5**: advs77942‐sup‐0005‐MovieS4.mov.

**Supporting File 6**: advs77942‐sup‐0006‐MovieS5.mov.

## Data Availability

The data that supports the findings of this study are available in the supplementary material of this article. The C–S–H dataset, CSH‐MLP, and README.md are publicly available on Figshare [85]. AIS Square: Datasets: https://www.aissquare.com/datasets/detail?pageType=datasets&name=CSH_dataset&id=340; Models: https://www.aissquare.com/models/detail?pageType=models&id=339.
