# Supplementary material for: Machine Learning Potential for Calcium Silicate Hydrates With Broad Compositional and Structural Diversity
Source: Adv Sci (Weinh). 2026 Sep 28:e77942. Online ahead of print. doi: 10.1002/advs.77942 (PMC13618771; doi:10.1002/advs.77942)
Supplement: Supplementary file 1 [file ADVS-9999-e77942-s004.docx]

Supplementary Information

Machine learning potential for calcium silicate hydrates with broad compositional and structural diversity

Yunjian Li^1,*,†^, Cheng Chen^1,†^, Roland J.-M. Pellenq^2^, Zongjin Li^3^, Jiaping Liu^4^,

^1^ State Key Laboratory of Climate Resilience for Coastal Cities, Department of Civil and Environmental Engineering, The Hong Kong University of Science and Technology, Hong Kong, People's Republic of China

^2^ Institut Européen des Membranes, CNRS, Université de Montpellier, 300 Avenue du Professeur Emile Jeanbrau, Montpellier, France

^3^ Faculty of Innovation Engineering, Macau University of Science and Technology, Macao, China

^4^ School of Materials Science and Engineering, Southeast University, Nanjing, People's Republic of China

† Yunjian Li and Cheng Chen contributed equally to this work.

*Corresponding author: Yunjian Li: E-mail: yunjianli@ust.hk

Supporting Movies:

Movie S1. csh_cp applied to the C–S–H model with a nanopore, front view.

Movie S2. csh_cp applied to the C–S–H model with a nanopore, left view.

Movie S3. ClayFF applied to the C–S–H model with a nanopore.

Movie S4. ReaxFF applied to the C–S–H model with a nanopore.

Movie S5. CSH-MLP applied to the C–S–H model with a nanopore.

1. **Supplementary figures and tables**

**Supplementary Figure 1.** Frequency distribution of the maximum standard deviation of atomic forces for C–S–H models with five different Ca/Si ratios.

**Supplementary Figure 2.** Frequency distribution of the maximum standard deviation of atomic forces for two C–S–H models made by the Pellenq method [1] and three tobermorite models in different basal spacings [2-4].

**Supplementary Figure 3. Distributions of absolute errors in the final training and test datasets.** Comparison between MLP and DFT results for (a) atomic energies, (b) x-component of forces, (c) y-component of forces, (d) z-component of forces.

**Supplementary Figure 4. Time evolution of the model pressure after deformation.** (a) and (b) Corresponding results obtained using the CSH-MLP and ERICA FF, respectively.

**Supplementary Figure 5. Assessment of deformation stability of the C–S–H model.** The maximum standard deviation of atomic forces calculated for C–S–H models made by the Pellenq’s method with different direction.

**Supplementary Figure 6. DFT validation of atomic forces predicted by CSH-MLP for C–S–H with Ca/Si = 1.47.** Comparison between the atomic forces predicted by CSH-MLP and the corresponding DFT reference values for the C–S–H model with Ca/Si = 1.47 used in the MLMD simulations.

**Supplementary Figure 7. Comparison of water and hydroxyl contents in C–S–H by different simulation methods.** Time evolution of (a) H_2_O/Si and (b) OH/Si ratios during 10 ps molecular dynamics simulations using AIMD, CSH-MLP, and ReaxFF [5, 6]. The calculations were performed on a C–S–H model with Ca/Si = 1.3, constructed from 11 Å tobermorite following the method proposed by Qomi et al [1, 3, 7]. CSH-MLP reproduces the AIMD evolution of water and hydroxyl contents more closely than ReaxFF.

**Supplementary Figure 8.** Snapshots of the C–S–H model after 100 ps NVT simulations using different force fields: (a) ReaxFF, (b) CSH-MLP, and (c) csh_cp. (d) Magnified view highlighting atomic clustering formation under csh_cp. (e) Same region showing hydrogen bonds (gray dashed lines) and O-O bonds (red solid lines). (f-h) Enlarged views of the C–S–H gel surface and pore water simulated with (f) ReaxFF, (g) CSH-MLP, and (h) csh_cp. The structures obtained with ReaxFF and CSH-MLP remain ordered and free of anomalies, whereas csh_cp produces extensive atomic clustering and abnormal calcium stacking on the C–S–H gel surface.

**Supplementary Figure 9. Comparison of the elastic constants of the C–S–H benchmark model using different computational methods:** (a) ClayFF, (b) ERICA FF, and (c) ReaxFF.

**Supplementary Figure 10. Relationship between C–S–H structural parameters and Ca/Si ratio. (a)** Relationship between Ca-OH/Ca ratio and Ca/Si ratio; **(b)** Relationship between Si-OH/Si ratio and Ca/Si ratio; **(c)** Relationship between mean chain length of silicate chains and Ca/Si ratio. Blue squares represent bulk C–S–H generated by pyCSH, and the orange dashed line represents the experimental data trend fitted by Casar et al. [8]

**Supplementary Table 1.** VRH approximation of elastic modulus and Poisson’s ratio. The unit of elastic tensors and moduli is [GPa]. The C–S–H benchmark model is a validation model for small systems. MLP denotes the CSH-MLP developed in this work; csh_cp is a machine-learning potential by Li et al. [9]; ReaxFF is the reactive force field by Manzano et al.[5]; and ClayFF and Erica FF are empirical force fields by Cygan et al. [10] and Valavi et al. [11], respectively.

|  | C–S–H benchmark model | | | | | | Large C–S–H model  (Ca/Si = 1.3) | | | | | Large C–S–H model  (Ca/Si = 1.5) | | | | | Large C–S–H model  (Ca/Si = 1.7) | | | | | Large C–S–H model  (Ca/Si = 1.9) | | | | |
| --- | --- | --- | --- | --- | --- | --- | --- | --- | --- | --- | --- | --- | --- | --- | --- | --- | --- | --- | --- | --- | --- | --- | --- | --- | --- | --- |
|  | DFT | MLP | csh_cp | ReaxFF | ClayFF | ERICA FF | MLP | csh_cp | ReaxFF | ClayFF | ERICA FF | MLP | csh_cp | ReaxFF | ClayFF | ERICA FF | MLP | csh_cp | ReaxFF | ClayFF | ERICA FF | MLP | csh_cp | ReaxFF | ClayFF | ERICA FF |
| Bulk modulus (V) | 56.02 | 56.21 | 56.88 | 54.01 | 52.61 | 59.11 | 47.61 | 46.19 | 54.09 | 41.59 | 52.28 | 54.50 | 53.12 | 49.93 | 43.24 | 58.14 | 51.71 | 54.36 | 44.75 | 43.01 | 56.23 | 49.14 | 54.60 | 45.53 | 39.41 | 54.17 |
| Bulk modulus (R) | 54.37 | 55.33 | 54.91 | 50.86 | 49.21 | 56.09 | 43.52 | 43.05 | 50.60 | 33.68 | 43.22 | 52.44 | 50.63 | 48.32 | 38.21 | 54.94 | 49.14 | 52.71 | 41.65 | 40.20 | 52.10 | 47.43 | 52.55 | 43.20 | 36.83 | 51.27 |
| Bulk modulus (H) | 55.19 | 55.77 | 55.89 | 52.43 | 50.91 | 57.60 | 45.57 | 44.62 | 52.34 | 37.64 | 47.75 | 53.47 | 51.87 | 49.12 | 40.73 | 56.54 | 50.43 | 53.54 | 43.20 | 41.60 | 54.16 | 48.29 | 53.58 | 44.37 | 38.12 | 52.72 |
| Shear modulus (V) | 25.30 | 27.04 | 27.82 | 19.69 | 25.91 | 33.34 | 25.34 | 25.36 | 27.20 | 23.11 | 31.38 | 26.53 | 26.03 | 25.30 | 21.81 | 31.65 | 23.51 | 25.99 | 22.97 | 20.48 | 30.45 | 23.09 | 23.63 | 21.63 | 17.68 | 27.77 |
| Shear modulus (R) | 23.50 | 22.29 | 24.64 | 12.99 | 22.19 | 29.89 | 22.42 | 22.78 | 25.18 | 17.44 | 25.51 | 24.15 | 24.22 | 24.13 | 17.41 | 27.98 | 20.18 | 24.39 | 21.67 | 17.64 | 27.54 | 20.61 | 22.50 | 20.06 | 15.41 | 24.75 |
| Shear modulus (H) | 24.40 | 24.66 | 26.23 | 16.34 | 24.05 | 31.62 | 23.88 | 24.07 | 26.19 | 20.27 | 28.45 | 25.34 | 25.13 | 24.72 | 19.61 | 29.82 | 21.85 | 25.19 | 22.32 | 19.06 | 29.00 | 21.85 | 23.06 | 20.85 | 16.54 | 26.26 |
| Young’s modulus (V) | 65.97 | 69.91 | 71.76 | 52.68 | 66.78 | 84.20 | 64.56 | 64.30 | 69.89 | 58.49 | 78.45 | 68.47 | 67.13 | 64.94 | 56.01 | 80.38 | 61.24 | 67.25 | 58.84 | 53.02 | 77.39 | 59.89 | 61.94 | 56.03 | 46.13 | 71.16 |
| Young’s modulus (R) | 61.62 | 58.95 | 64.30 | 35.92 | 57.87 | 76.14 | 57.40 | 58.09 | 64.78 | 44.61 | 63.96 | 62.81 | 62.67 | 62.06 | 45.33 | 71.75 | 53.26 | 63.39 | 55.40 | 46.17 | 70.24 | 54.01 | 59.07 | 52.12 | 40.56 | 63.96 |
| Young’s modulus (H) | 63.80 | 64.49 | 68.04 | 44.42 | 62.34 | 80.18 | 60.98 | 61.20 | 67.34 | 51.56 | 71.20 | 65.65 | 64.90 | 63.50 | 50.69 | 76.08 | 57.27 | 65.32 | 57.12 | 49.61 | 73.82 | 56.96 | 60.51 | 54.08 | 43.36 | 67.57 |
| Poisson’s ratio (V) | 0.30 | 0.29 | 0.29 | 0.34 | 0.29 | 0.26 | 0.27 | 0.27 | 0.28 | 0.27 | 0.25 | 0.29 | 0.29 | 0.28 | 0.28 | 0.27 | 0.30 | 0.29 | 0.28 | 0.29 | 0.27 | 0.30 | 0.31 | 0.29 | 0.30 | 0.28 |
| Poisson’s ratio (R) | 0.31 | 0.32 | 0.30 | 0.38 | 0.30 | 0.27 | 0.28 | 0.28 | 0.29 | 0.28 | 0.25 | 0.30 | 0.29 | 0.29 | 0.30 | 0.28 | 0.32 | 0.30 | 0.28 | 0.31 | 0.28 | 0.31 | 0.31 | 0.30 | 0.32 | 0.29 |
| Poisson’s ratio (H) | 0.31 | 0.31 | 0.30 | 0.36 | 0.30 | 0.27 | 0.28 | 0.27 | 0.29 | 0.27 | 0.25 | 0.30 | 0.29 | 0.28 | 0.29 | 0.28 | 0.31 | 0.30 | 0.28 | 0.30 | 0.27 | 0.30 | 0.31 | 0.30 | 0.31 | 0.29 |

**Supplementary Table 2.** Elastic constants for different Ca/Si. The unit of elastic constants are GPa. The C–S–H benchmark model is a validation model for small systems. MLP denotes the CSH-MLP developed in this work; csh_cp is a machine-learning potential by Li et al. [9]; ReaxFF is the reactive force field by Manzano et al. [5]; and ClayFF and Erica FF are empirical force fields by Cygan et al. [10] and Valavi et al. [11], respectively.

|  | C–S–H benchmark model | | | | | | Large C–S–H model (Ca/Si = 1.3) | | | | | Large C–S–H model (Ca/Si = 1.5) | | | | | Large C–S–H model (Ca/Si = 1.7) | | | | | Large C–S–H model (Ca/Si = 1.9) | | | | |
| --- | --- | --- | --- | --- | --- | --- | --- | --- | --- | --- | --- | --- | --- | --- | --- | --- | --- | --- | --- | --- | --- | --- | --- | --- | --- | --- |
|  | DFT | MLP | csh_cp | ReaxFF | ClayFF | ERICA FF | MLP | csh_cp | ReaxFF | ClayFF | ERICA FF | MLP | csh_cp | ReaxFF | ClayFF | ERICA FF | MLP | csh_cp | ReaxFF | ClayFF | ERICA FF | MLP | csh_cp | ReaxFF | ClayFF | ERICA FF |
| **C_11_** | 105.14 | 106.37 | 101.15 | 65.42 | 89.46 | 112.52 | 99.12 | 96.11 | 102.36 | 94.57 | 124.38 | 105.94 | 101.22 | 95.87 | 92.72 | 117.77 | 100.87 | 99.26 | 90.18 | 85.56 | 111.98 | 95.15 | 95.75 | 85.11 | 74.58 | 105.28 |
| **C_22_** | 96.60 | 98.26 | 110.67 | 91.38 | 114.98 | 124.86 | 96.71 | 91.86 | 105.11 | 98.88 | 121.43 | 100.02 | 95.43 | 93.02 | 88.50 | 117.54 | 94.64 | 97.07 | 87.15 | 81.23 | 114.61 | 91.46 | 96.51 | 87.81 | 74.59 | 106.55 |
| **C_33_** | 81.49 | 82.94 | 80.33 | 93.27 | 68.84 | 88.45 | 60.39 | 60.28 | 70.77 | 42.07 | 58.79 | 76.04 | 72.74 | 74.87 | 51.35 | 83.59 | 72.33 | 76.02 | 58.98 | 54.48 | 74.94 | 68.44 | 71.64 | 62.89 | 49.97 | 75.88 |
| **C_12_** | 43.93 | 38.53 | 40.65 | 46.41 | 40.54 | 46.99 | 37.92 | 35.51 | 42.97 | 34.38 | 45.67 | 42.48 | 45.31 | 38.22 | 35.66 | 47.02 | 40.57 | 42.43 | 36.09 | 35.47 | 47.27 | 37.63 | 45.19 | 35.87 | 33.35 | 46.46 |
| **C_13_** | 35.24 | 32.53 | 31.61 | 36.87 | 27.04 | 26.77 | 23.50 | 24.25 | 29.36 | 18.01 | 18.84 | 30.64 | 31.07 | 29.21 | 21.86 | 27.34 | 28.52 | 32.61 | 24.72 | 22.28 | 27.02 | 27.70 | 34.47 | 25.20 | 22.20 | 26.84 |
| **C_23_** | 31.31 | 38.11 | 37.60 | 34.71 | 32.55 | 29.31 | 24.70 | 23.96 | 31.96 | 17.01 | 18.45 | 31.12 | 27.95 | 25.38 | 20.78 | 27.83 | 29.71 | 33.42 | 22.40 | 25.16 | 27.97 | 28.29 | 34.08 | 25.91 | 22.21 | 26.62 |
| **C_44_** | 21.80 | 21.28 | 25.39 | 12.39 | 20.85 | 24.31 | 18.27 | 18.36 | 20.46 | 13.17 | 19.35 | 18.73 | 20.96 | 19.86 | 12.17 | 22.46 | 18.33 | 23.10 | 17.76 | 13.86 | 23.92 | 16.92 | 20.25 | 15.23 | 12.29 | 18.90 |
| **C_55_** | 21.96 | 20.05 | 22.10 | 13.81 | 17.67 | 22.83 | 16.33 | 17.93 | 22.74 | 10.56 | 17.46 | 19.44 | 19.83 | 21.56 | 11.92 | 19.59 | 12.27 | 21.03 | 19.12 | 12.17 | 20.10 | 14.66 | 19.30 | 16.50 | 10.44 | 17.75 |
| **C_66_** | 25.17 | 34.41 | 30.85 | 28.24 | 33.34 | 45.33 | 35.38 | 35.64 | 34.81 | 36.42 | 46.21 | 35.21 | 34.37 | 28.11 | 33.53 | 43.99 | 30.58 | 31.17 | 26.94 | 30.24 | 41.83 | 30.07 | 28.53 | 26.83 | 25.20 | 39.62 |
| **C_14_** | 0.49 | -1.81 | -3.56 | -0.74 | 3.72 | 0.51 | 0.47 | -2.22 | 0.09 | 0.01 | 1.88 | -1.27 | -0.17 | -2.49 | -2.36 | -1.62 | -0.65 | 0.67 | -0.88 | -1.80 | -0.34 | 0.58 | -1.60 | 1.12 | -0.61 | -1.08 |
| **C_15_** | -2.23 | 1.27 | -4.15 | -0.59 | -2.82 | -1.22 | -0.78 | -0.51 | 0.46 | -1.63 | -0.59 | -1.07 | -2.23 | 0.63 | -1.62 | -0.54 | 0.81 | 1.65 | 0.90 | -0.19 | -0.53 | 0.17 | -0.71 | 1.20 | -1.35 | 0.55 |
| **C_16_** | 5.64 | 0.05 | 0.21 | 1.14 | -0.70 | 0.50 | -2.22 | -1.33 | -2.35 | -3.45 | -4.37 | 0.24 | 0.64 | 0.14 | -4.14 | -3.17 | 0.86 | 2.27 | -2.43 | -0.41 | -2.52 | 0.18 | 3.05 | -2.73 | 0.44 | -0.17 |
| **C_24_** | -1.64 | -6.57 | 0.32 | -3.56 | -2.27 | 0.40 | 2.20 | 0.49 | -0.51 | 0.73 | -0.07 | -1.44 | -0.41 | -1.79 | 0.41 | -0.45 | -1.95 | 0.76 | -1.58 | -0.16 | -0.63 | 0.52 | 1.29 | 1.73 | 1.51 | 0.18 |
| **C_25_** | -2.23 | 6.59 | 6.14 | -2.43 | 1.29 | -0.69 | -2.45 | 0.15 | 0.57 | -0.90 | -1.57 | -1.90 | -3.91 | -0.96 | -1.52 | -0.46 | -0.03 | 2.87 | 0.15 | -0.20 | 0.96 | -1.65 | 0.56 | 1.93 | -1.90 | -0.12 |
| **C_26_** | 5.64 | 1.89 | -0.34 | -7.52 | 2.39 | -2.16 | -2.51 | -2.05 | -5.46 | -2.86 | -7.55 | -1.21 | -1.11 | -1.50 | -4.40 | -6.14 | 2.76 | -0.47 | -2.09 | -1.04 | -5.46 | 1.13 | 1.12 | -3.01 | -2.65 | -5.04 |
| **C_34_** | 2.05 | 1.75 | -0.10 | -6.77 | -2.66 | -5.51 | 1.83 | -0.53 | 2.02 | -0.88 | 0.72 | 0.13 | -0.38 | -1.21 | -2.68 | -1.30 | -2.54 | 0.18 | -2.00 | 0.37 | -0.63 | -0.10 | 1.00 | 0.21 | -1.11 | 1.22 |
| **C_35_** | 0.87 | -2.90 | 6.18 | -4.10 | -0.10 | -4.25 | -2.74 | -1.99 | 1.65 | 0.21 | 0.66 | -1.29 | -0.33 | -1.76 | 0.02 | -0.33 | 4.76 | 2.17 | 0.88 | 0.71 | 2.56 | 0.29 | -1.22 | 1.67 | -0.94 | -0.36 |
| **C_36_** | -7.47 | 0.91 | 1.11 | -5.09 | -1.30 | -1.37 | -0.32 | -1.99 | -4.48 | -0.45 | -2.30 | -1.70 | -0.54 | -3.78 | -1.19 | -2.13 | 1.28 | 0.95 | -2.11 | -1.18 | -2.17 | -0.20 | 1.33 | -2.65 | -0.33 | -1.40 |
| **C_45_** | -4.67 | -0.27 | -5.52 | -5.68 | -5.44 | -0.87 | -1.42 | -2.48 | -2.50 | -1.38 | -3.01 | -1.96 | -3.11 | -1.59 | -1.93 | -2.27 | -0.05 | -3.79 | -0.95 | -1.75 | -2.72 | -2.67 | -1.53 | 0.16 | -1.31 | -0.73 |
| **C_46_** | -0.45 | -5.33 | -3.03 | 2.11 | -0.93 | -0.14 | -1.34 | -1.47 | -0.66 | -1.80 | -1.71 | -1.38 | -1.99 | -2.07 | -1.00 | -0.52 | 0.78 | 1.20 | -0.97 | -0.30 | -0.56 | -0.79 | -0.56 | 0.61 | -0.36 | -0.51 |
| **C_56_** | 1.57 | 10.73 | 7.32 | 6.06 | 6.38 | 6.92 | 4.29 | 4.15 | 5.97 | 2.63 | 4.31 | 5.33 | 3.68 | 3.67 | 2.18 | 4.90 | 2.75 | 5.36 | 4.04 | 2.57 | 4.17 | 2.90 | 4.29 | 3.19 | 1.53 | 3.00 |

2. **Supplementary methods**

**2.1 Non-equilibrium and metastable configurations sampling from the MD trajectory**

Non-equilibrium and metastable configurations sampling from the MD trajectory was performed using a strictly stage-resolved strategy aligned with the degree of equilibration of the system. In the initial structural relaxation stage, the system was equilibrated under the NVT ensemble by progressively releasing atomic degrees of freedom: first allowing only water molecules to move, then including hydroxyl groups and hydroxide ions, and finally releasing calcium atoms. Each sub-stage was run for 50,000 steps with a timestep of 0.05 fs. During this phase, configurations were recorded every 10,000 steps (corresponding to 0.5 ps per frame), resulting in a total of 15 configurations. After this pre-equilibration, the system underwent multiple rounds of energy minimization with alternating fixed-cell and variable-cell relaxations, during which 3 configurations were recorded. Subsequently, a short core–shell equilibration was carried out under a polarizable core–shell model; no trajectory frames were saved at this stage, as it was solely intended to stabilize the polarization degrees of freedom.

The system then entered the structural equilibration and heating–quenching cycles, consisting of multiple NPT and NVT simulations, including NPT equilibration from 1 K to 300 K, NVT heating from 300 K to 700 K, extended equilibration at 700 K, and subsequent cooling back to room temperature. Depending on the equilibration stage and simulation objective, trajectories were recorded every 1,000, 10,000, or 50,000 steps (corresponding to time intervals of approximately 0.2–10 ps) to systematically capture the structural evolution of C–S–H under varying temperature and pressure conditions. This stage yielded a total of 335 configurations. The final production run was conducted under the NPT ensemble at 298.15 K and 1 atm with a timestep of 0.5 fs and a total simulation time of 1 ns. Trajectories were recorded every 50,000 steps (25 ps); including the initial configuration, this stage produced 41 configurations. In total, the complete equilibration and production workflow for one MD equilibration trajectories generated 394 C–S–H configurations.

We selected 100 MD-equilibration trajectories from the total set of 1,921 trajectories, which would yield 100 × 394 = 39,400 configurations in principle. However, it should be pointed out that, during the initial structural relaxation stage, some structures are not well optimized, which would lead to subsequent DFT calculations failed or unconverging, thus these unphysical configurations should be precluded. As a result, a total of 39,057 configurations were successfully labeled and retained in the final dataset.

3. **Coordination number of Ca in confined spaces**

**Supplementary Table 3.** Ca coordination number (CN) in confined spaces with different sizes *D*.

| Confined space size *D* (Å) | Distribution of Ca CN | | | | Total number | Mean CN |
| --- | --- | --- | --- | --- | --- | --- |
|  | **5** | **6** | **7** | **8** |  |  |
| 8 | 15 | 92 | 51 | 2 | 160 | 6.25 |
| 10 | 8 | 91 | 56 | 5 | 160 | 6.36 |
| 14 | 7 | 89 | 58 | 6 | 160 | 6.39 |
| 29 | 5 | 72 | 75 | 8 | 160 | 6.54 |

As illustrated in Supplementary Figure 11, the C–S–H models employed to investigate the coordination number of Ca in confined spaces have in-plane dimensions of approximately 45.4 Å × 59.0 Å, with confinement sizes *D* of approximately 8 Å, 10 Å, 14 Å, and 29 Å, respectively. The CSH-MLP was utilized to perform both equilibration and production simulations for the C–S–H models, with a time step of 1 fs. The equilibration stage consisted of two annealing–quenching cycles, each lasting 400 ps, during which the temperature was controlled between 300 K and 700 K. Following the quenching process, an additional 200 ps NPT equilibration was conducted at 300 K to ensure structural stabilization. During the production stage, the system was maintained at 300 K, and a 1 ns production run was performed to collect statistical data for subsequent analysis.

**Supplementary Figure 11.** Schematic illustration of the confined C–S–H system. The C–S–H model has dimensions of 45.4 Å × 59.0 Å × 43.9 Å, and the confined space size is *D* ≈ 14 Å.

# **Reference**

[1] R.J.M. Pellenq, A. Kushima, R. Shahsavari, K.J. Van Vliet, M.J. Buehler, S. Yip, F.-J. Ulm, A realistic molecular model of cement hydrates, Proceedings of the National Academy of Sciences, 106 (2009) 16102–16107, 10.1073/pnas.0902180106.

[2] S. Merlino, E. Bonaccorsi, T. Armbruster, The real structures of clinotobermorite and tobermorite 9 A: OD character, polytypes, and structural relationships, European Journal of Mineralogy, 12 (2000) 411–429.

[3] S. Merlino, E. Bonaccorsi, T. Armbruster, The real structure of tobermorite 11A: normal and anomalous forms, OD character and polytypic modifications, European Journal of Mineralogy, 13 (2001) 577–590.

[4] E. Bonaccorsi, S. Merlino, A.R. Kampf, The Crystal Structure of Tobermorite 14 Å (Plombierite), a C–S–H Phase, Journal of the American Ceramic Society, 88 (2005) 505–512, <https://doi.org/10.1111/j.1551-2916.2005.00116.x>.

[5] H. Manzano, R.J. Pellenq, F.-J. Ulm, M.J. Buehler, A.C. Van Duin, Hydration of calcium oxide surface predicted by reactive force field molecular dynamics, Langmuir, 28 (2012) 4187–4197.

[6] H. Manzano, S. Moeini, F. Marinelli, A.C. Van Duin, F.-J. Ulm, R.J.-M. Pellenq, Confined water dissociation in microporous defective silicates: mechanism, dipole distribution, and impact on substrate properties, Journal of the American Chemical Society, 134 (2012) 2208–2215.

[7] M.J. Abdolhosseini Qomi, K.J. Krakowiak, M. Bauchy, K.L. Stewart, R. Shahsavari, D. Jagannathan, D.B. Brommer, A. Baronnet, M.J. Buehler, S. Yip, F.J. Ulm, K.J. Van Vliet, R.J.M. Pellenq, Combinatorial molecular optimization of cement hydrates, Nature Communications, 5 (2014) 4960, 10.1038/ncomms5960.

[8] Z. Casar, J. López-Zorrilla, H. Manzano, E. Duque-Redondo, A.K. Mohamed, K. Scrivener, P. Bowen, pyCSH: Automated atomic-level structure generation of bulk C-S-H and investigation of their intrinsic properties, Cement and Concrete Research, 183 (2024) 107593, <https://doi.org/10.1016/j.cemconres.2024.107593>.

[9] W. Li, Y. Zhou, L. Ding, P. Lv, Y. Su, R. Wang, C. Miao, A deep learning-based potential developed for calcium silicate hydrates with both high accuracy and efficiency, Journal of Sustainable Cement-Based Materials, 12 (2023) 1335–1346, 10.1080/21650373.2023.2219251.

[10] R.T. Cygan, J.-J. Liang, A.G. Kalinichev, Molecular models of hydroxide, oxyhydroxide, and clay phases and the development of a general force field, The Journal of Physical Chemistry B, 108 (2004) 1255–1266.

[11] M. Valavi, Z. Casar, A. Kunhi Mohamed, P. Bowen, S. Galmarini, Molecular dynamic simulations of cementitious systems using a newly developed force field suite ERICA FF, Cement and Concrete Research, 154 (2022) 106712, <https://doi.org/10.1016/j.cemconres.2022.106712>.
